# Single-cell transcriptome analysis reveals the malignant characteristics of tumour cells and the immunosuppressive landscape in HER2-positive inflammatory breast cancer

**DOI:** 10.1186/s13046-025-03454-z

**Published:** 2025-07-08

**Authors:** Juan Huang, Yongwei Zhu, Wenjing Zeng, Yulong Zhang, Weizhi Xia, Fan Xia, Liyu Liu, Kuansong Wang, Yidi Guan, Taohong Shen, Bingjian Jiang, Lunquan Sun, Ayong Cao, Shouman Wang, Zhi Li

**Affiliations:** 1 Department of Breast Surgery, Xiangya Hospital, Central South University, Changsha, China; 2 Multidisciplinary Breast Cancer Center, Xiangya Hospital, Central South University, Changsha, China; 3 Clinical Research Center For Breast Cancer In Hunan Province, Xiangya Hospital, Central South University, Changsha, China; 4 Xiangya Cancer Center, Xiangya Hospital, Central South University, Changsha, China; 5 Department of Neurosurgery, Xiangya Hospital, Central South University, Changsha, China; 6 Department of Respiratory, The Sixth Clinical Medical College of Xinjiang Medical University, Urumqi, China; 7 The Second Affiliated Hospital of Xinjiang Medical University, Urumqi, China; 8 Key Laboratory of Molecular Radiation Oncology Hunan Province, Changsha, China; 9 Institute of Cancer Research, National Clinical Research Center for Geriatric Disorders (Xiangya), Xiangya Hospital, Central South University, Changsha, China; 10 Department of Pathology, School of Basic Medical Sciences, Central South University, Changsha, Hunan China; 11 Huarong County People’s Hospital, Hunan, China; 12 Department of Breast and Thyroid Surgery, The People’s Hospital of Xiangxi Autonomous Prefecture, Jishou, Hunan China; 13 Key Laboratory of Breast Cancer, Department of Breast Surgery, Fudan University Shanghai Cancer Center, Shanghai, China

**Keywords:** IBC, HER2, ScRNA, TME, Immunosuppression, PTN, TNF

## Abstract

**Background:**

Inflammatory breast cancer (IBC), of which HER2 + is the predominant subtype, is extremely aggressive and difficult to treat. Previous studies have suggested that targeting the tumour microenvironment (TME) may provide new directions for IBC diagnosis and treatment.

**Methods:**

In this study, we used single-cell transcriptome technology (scRNA-seq) to investigate the molecular features of the TME of HER2 + IBC patients and performed a comprehensive and detailed comparison of the cellular components and molecular phenotypes of the TME between IBC patients and noninflammatory breast cancer (nIBC) patients to elucidate the cell types that are specifically enriched in the TME of IBC patients, as well as the molecular features that are responsible for the preferential remodelling of the cellular functional state in the TME.

**Results:**

A total of 15,832 cells, including epithelial cells, endothelial cells, stromal cells, T cells, B cells, antibody secreting cells (ASCs) and myeloid cells, were obtained from tumour tissues from 3 HER2 + IBC patients for scRNA analysis. By comparing the TME with that of HER2 + nIBC patients in a public database, we found that the TME of HER2 + IBC patients had a greater level of lymphocyte infiltration than that of nIBC patients did, and an especially significant enrichment of ASCs (mainly plasmablasts or plasma cells). In the TME of HER2 + IBC patients, tumour-infiltrating T cells exhibited a dual molecular phenotype of high activation and high exhaustion, with tumour-infiltrating B cells preferring the extrafollicular developmental pathway, and tumour-infiltrating myeloid and mesenchymal cells exhibiting a greater immunosuppressive status. By performing a cellular interaction analysis, we revealed that PTN molecules were significantly overexpressed in HER2 + IBC tumour cells and that the cellular interactions mediated by these molecules were strongly correlated with the functional polarisation of the cellular components in the TME. We observed that HER2 + IBC tumour cells have an active interferon response and epithelial mesenchymal transition (EMT) signalling, and that their malignant process is strongly correlated with the inflammatory response. Moreover, we found that HER2 + IBC tumour-infiltrating B cells promoted necroptosis of endothelial cells through high expression of TNF, thus promoting inflammatory responses.

**Conclusion:**

We found a strong correlation between high expression of PTN molecules in HER2 + IBC tumour cells and their highly invasive characteristics and highly immunosuppressive TME. These results suggest that HER2 + IBC tumour cells can promote an inflammatory response by upregulating the expression of TNF molecules in B cells via PTN molecules and that the enhanced inflammatory response in turn promotes tumour progression, a malignant cycle that shapes a more immunosuppressive TME. Therefore, diagnostic and therapeutic strategies targeting the PTN-TNF molecular axis may have considerable potential for development in HER2 + IBC patients.

**Supplementary Information:**

The online version contains supplementary material available at 10.1186/s13046-025-03454-z.

## Background

IBC is a rare and highly aggressive subtype of breast cancer [1]. It is characterized by a rapidly progressive course, erythema and orange peel-like changes in the skin of the breast [2] without a distinct palpable mass but pathologically presents more features of vascular cancer emboli [3]. The propensity of IBC for lymph node and distant metastases [4] and challenges in early detection [3] contribute to its poor prognosis. Currently, the molecular classification and treatment of IBC are modelled after those for nIBC [5], yet notable differences exist in subtype prevalence (e.g., HR: 30% in IBC vs. 60%–80% in nIBC; HER2-positive: 40% in IBC vs. 25% in nIBC; triple-negative: 30% in IBC vs. 10%–15% in nIBC) and treatment efficacy [6]. HER2 + is the predominant subtype of IBC [1], and while dual-targeted therapies have improved survival [7], the 5-year survival rate remains below 50%, which is far lower than that of nIBC [6]. Traditional treatments, including surgery, radiotherapy, chemotherapy, and endocrine therapies, often yield poor responses in IBC patients [6]. The rapid deterioration of IBC, often without typical symptoms [2], makes it particularly challenging to diagnose and treat [3]. Research has thus far focused on genetic diversity to identify potential biomarkers [8, 9], but the development of new therapies is slow due to case rarity and difficulties in clinical trials [10]. Therefore, there is an urgent need for interdisciplinary collaboration and precision medicine to elucidate the pathogenic mechanisms and identify potential molecular targets for IBC.

Recent preclinical and clinical research has provided new molecular insights into IBC pathogenesis [11]. In 2013, the World Alliance for IBC identified a distinct molecular subtype characterized by 79 feature genes with attenuated TGF-β signalling that could predict patient outcomes [8]. Genomic studies have highlighted common mutations in IBC, such as MYC (32%), PIK3CA (28%), HER2 (26%), and FGFR1 (17%) amplifications and TP53 (62%), BRCA2 (15%), and PTEN (15%) mutations [12]. These molecular alterations drive cancer cell proliferation and survival and modulate immune regulation within the TME [10]. The emergence of immunotherapy has introduced new treatment possibilities, with trials exploring immune checkpoint inhibitors such as PD-L1 and CTLA-4 alone or in combination with other therapies [13, 14]. However, the efficacy of immunotherapy in IBC remains uncertain because of limited clinical data [14]. The IBC immune microenvironment is characterized by preexisting active immunity and immune escape under various inhibitory factors [14], and the effectiveness of checkpoint inhibitors in overcoming this suppression is still debated [14]. Understanding the mechanisms behind the immune-suppressive environment in IBC is crucial for developing precise immunotherapy strategies.

Understanding the TME of HER2 + IBC patients has largely relied on histopathological features and traditional genomic or transcriptomic sequencing [10], and exploration of the diversity and complexity of cell interactions is lacking [15]. scRNA analysis offers high-resolution molecular profiles, revealing complex interactions among cell subpopulations and treatment response heterogeneity [16, 17]. Previous studies have used single-cell atlases to reveal breast epithelial cell subpopulations and their relationships with specific breast cancer subtypes [18, 19]. High-resolution single-cell studies have also revealed spatial heterogeneity in the TME [20]. These advances have significantly enhanced our understanding of the cellular functions and interactions within the TME and support the development of targeted and immunotherapy strategies. Therefore, the application of scRNA-seq to study the TME of IBC will deepen our understanding of its highly immune-suppressive nature and its ability to invade blood vessels.

In this study, we used scRNA-seq to reveal the molecular features of the TME of HER2 + IBC and performed a comprehensive and detailed comparison of the molecular phenotypes of tumour cells, mesenchymal cells, myeloid cells, T cells, B cells, and antibody-secreting cells between IBC and nIBC patients. A comparison of the cellular components revealed that the cell types specifically enriched in the TME of HER2 + IBC patients were mainly T cells, B cells and antibody-secreting cells. Through the assessment and comparison of cellular functional states, we observed that immune cells in the TME of HER2 + IBC patients exhibit a more dysfunctional state, with T cells exhibiting a dual molecular phenotype of high activation and high suppression, B cells exhibiting a preference for extrafollicular development, and myeloids and mesenchymal cells exhibiting a stronger immunosuppressive state. Through a systematic comparison of cellular interactions, we observed that HER2 + IBC tumour cells have specific PTN ligand molecule-mediated interactions that promote a tumour-suppressive microenvironment. Moreover, we observed specific high expression of TNF signalling in IBC tumour-infiltrating B cells, which promoted inflammatory responses by mediating necroptosis through interactions with endothelial cells. In conclusion, our integrative analysis targeting the TME of HER2 + IBC and nIBC patients revealed a stronger immunosuppressive molecular signature in HER2 + IBC patients, suggesting that cellular interaction pathways mediated by the PTN-TNF molecular axis, which are specific to HER2 + IBC tumours, are potentially important therapeutic targets.

## Methods

### Collection of tumour samples from patients

In accordance with international standards and the sampling protocols of Xiangya Hospital, Central South University, we performed core needle biopsies on tumour tissues from three IBC patients and recorded their clinical data. Informed consent was obtained from each patient prior to the procedure. The biopsies were performed under imaging guidance to ensure precision, following strict and normative standards. After collection, the samples were promptly immersed in appropriate fixatives for preservation. The detailed clinical characteristics of these samples are listed in Supplementary Table S1.

### Collection of public single-cell sata

Single-cell transcriptomic data for normal breast tissue were sourced from the Gene Expression Omnibus (GEO) database, mainly from the datasets GSE161529 [19, 21] and GSE164898 [18]. To ensure compatibility in terms of age, parity, and menopausal status, we selected a final cohort of five samples for inclusion in our study on normal breast tissue. Additionally, single-cell transcriptomic data from nine HER2 + nIBC patients were also retrieved from GEO, with the primary sources being GSE176078 [20] and GSE161529 [19]. The relevant clinical characteristics of these samples are detailed in Supplementary Table S1.

### Single-cell transcriptomic capture

Biopsy samples were transported to the laboratory within one hour in chilled containers. In the laboratory, nonadipose tissue was cut into approximately 1 mm^3^ pieces on ice and transferred into 1.5 ml tubes containing Dulbecco’s modified Eagle’s medium (Thermo Fisher Scientific). The tissue was minced using ophthalmic scissors and washed with 1 × phosphate-buffered saline (PBS) to remove any circulating immune cells. The washed tissue was then transferred to 15 ml tubes, dissociated using a tissue dissociation kit with mixed enzymes (Miltenyi Biotec, cat. no. 130–110-203), and incubated for 30 min at 37 °C with agitation. The resulting cell suspension was filtered through a 70 µm mesh and centrifuged at 400 × g at room temperature (20–22 °C) for 5 min. The supernatant was discarded, and the cell pellet was resuspended in red blood cell lysis buffer (Solarbio) and incubated at room temperature (20–22 °C) for 5 min to lyse red blood cells. The suspension was then filtered through a 40 µm mesh. After two washes with 1 × PBS, the tissue was resuspended in 1 × PBS. Cell viability was evaluated using Trypan blue (Sigma) staining under a microscope. For samples with a high percentage of dead cells, additional processing was performed using a dead cell removal kit (Miltenyi Biotec, cat. no. 130–090-101).

Single-cell capture and sequencing were carried out using the GemCode single-cell platform. The GemCode gel beads, chip, and library preparation kits (10 × Genomics, PN-120236) were used following the manufacturer’s instructions. Single-cell suspensions were loaded into channels with a target of 8,000 cells per sample, with careful volume control. This step enables single-cell labelling, mRNA capture, reverse transcription, cDNA recovery, amplification, and library construction. The libraries were sequenced on the Illumina NovaSeq 6000 platform using paired-end 150 bp sequencing.

### Single-cell transcriptome data analysis

Raw single-cell RNA sequencing data were processed and analysed using CellRanger (v.6.1.1, 10 × Genomics), referencing the GRCh38 human genome and its annotations. Each read was tagged with unique molecular identifiers (UMIs) to distinguish individual transcripts. A single-cell by gene expression matrix was constructed by counting UMIs per gene for each barcode-labelled cell.

Data analysis was performed using the R package Seurat (v4.0.4, http://satijalab.org/seurat/) [22]. Low-quality cells were filtered on the basis of the following criteria: (1) each cell needed to have more than 200 and fewer than 7,500 expressed genes; (2) the proportion of UMIs mapping to mitochondrial genes had to be under 25%. Doublets were removed using DoubletFinder v3 [23]. Additionally, the cells were assessed using dissociation-related gene scores (Supplementary Table S2), and the top 3% with the highest scores were excluded to account for dissociation artefacts. Cells with detection rates less than 40% for housekeeping genes and greater than 5% for red blood cell-related genes were also discarded. Dimensionality reduction and clustering of the filtered expression matrix were conducted using Seurat. The “vst” method identified the top 2,000 variable features per dataset. Harmony [24] was used for batch effect correction across datasets. The ScaleData function was used to perform linear scaling of the variable features, and principal component analysis (PCA) was applied to the scaled data. The statistical significance of the PCA scores was evaluated with the JackStraw function. Clustering was performed using the first 30 principal components at a resolution of 0.8, and the cell clusters were visualized using uniform manifold approximation and projection (UMAP). Marker genes specific to each cluster were identified with the FindAllMarkers function. On the basis of the expression of marker genes, the cells were classified into seven primary types: epithelial cells, endothelial cells, stromal cells, T cells, B cells, antibody-secreting cells, and myeloid cells.

### Identification of malignant cells

To differentiate malignant from nonmalignant epithelial cells, we utilized three complementary methods for a thorough evaluation of the malignancy of epithelial cells from each sample. Initially, epithelial cells were isolated on the basis of high expression markers (EPCAM, KRT5, KRT14, KRT18, and KRT19). These cells were subsequently reclustered using Seurat [22], resulting in 26 subpopulations. Each subpopulation’s tissue origin was analysed: those with more than 10% normal breast tissue were categorized as normal epithelial cells, those with less than 1% normal tissue were categorized as malignant epithelial cells, and the rest were categorized as mixed-state epithelial cells. To validate the results of malignant cell identification, copy number variation (CNV) levels were evaluated with the R packages inferCNV (https://github.com/broadinstitute/inferCNV) and CopyKat [25], with normal breast tissue-derived epithelial cells used as controls and tumour-derived epithelial cells used as the experimental group.

### Pathway enrichment analysis

Differential transcriptomic features between cell subtypes were compared using several approaches as follows, with a focus on differentially expressed genes and pathway enrichment:

1. Gene Ontology (GO) enrichment: Differentially expressed genes were identified with FindAllMarkers, applying a threshold of log2-fold change > 0.25 and adjusted *P* value < 0.05. Pathway enrichment for highly expressed genes in subgroups was analysed using clusterProfiler [26], referencing Gene Ontology biological process (GOBP) (https://www.gsea-msigdb.org/gsea/msigdb/human/collections.jsp#C5) and Kyoto Encyclopedia of Genes and Genomes (KEGG) (https://www.gsea-msigdb.org/gsea/msigdb/human/genesets.jsp?collection=CP:KEGG_LEGACY).
2. Gene set enrichment analysis (GSEA): Expression differences between subgroups were evaluated with the limma package [27, 28], which uses log2-fold change and -log10-adjusted P values for ranking. Enrichment analysis of gene sets, focusing on hallmark sets from MsigDB (https://www.gsea-msigdb.org/gsea/msigdb/human/collections.jsp#H), was conducted with clusterProfiler.
3. Feature gene set scoring: AUCell (https://bioconductor.org/packages/release/bioc/html/AUCell.html) was used to score gene sets related to tumour malignancy [29, 30] (14 gene sets) and the TME [31] (29 gene sets) for each cell.

### Cell trajectory analysis

Trajectory analysis of cells in various states was performed using Monocle2 (http://cole-trapnell-lab.github.io/monocle-release/) [32] to explore transcriptomic changes during differentiation or activation. Differentially expressed genes were identified with FindAllMarkers and filtered for log2-fold change > 0.5 and adjusted P value < 0.05. Dimensionality reduction was executed with the reduceDimension function, setting max_components to 2 and the DDRTree method. The cells were ordered with orderCells using default parameters. Differentiation potential was assessed with CytoTRACE (https://cytotrace.stanford.edu/) [33], where higher scores indicate greater differentiation potential.

### Cell communication analysis

Cell communication patterns were analysed using the CellChat package [34] to identify significant cellular interactions and receptor‒ligand pairs. The single-cell expression matrix was projected onto the human receptor‒ligand interaction network using the projectData function. Interactions were inferred with computeCommunProb, computeCommunProbPathway, and aggregateNet functions under default settings. The results were visualized using the netVisual_circle, netVisual_bubble, netVisual_aggregate, and netAnalysis_signalingRole_heatmap functions.

### Prognostic analysis of data from The Cancer Genome Atlas (TCGA)

We explored and visualized the expression and prognostic significance of SOX4 across 33 cancer types using GEPIA2 [35] (http://gepia2.cancer-pku.cn/#index). Differences in SOX4 expression between tumour and normal tissues are presented in scatter plots, and heatmaps illustrate the correlation between SOX4 levels and overall survival in cancer patients.

### Correlation analysis of gene expression

Pearson correlation coefficients were used for single gene correlations, which were visualized with ggscatterhist. For correlations between one gene and multiple genes, AUCell scores for gene sets were calculated, and Pearson correlations were evaluated and visualized. For multiple gene correlations, gene sets were scored with AUCell and analysed similarly for correlation and visualization.

### Multiplex immunofluorescence

Formalin-fixed, paraffin-embedded (FFPE) blocks from IBC and nIBC samples were sectioned into 4 µm sections. The sections were incubated at 65–70 °C for 1.5 h, followed by a 15-min xylene treatment (repeated twice), and then subjected to a graded ethanol series (100% × 2: 3 min, 95%: 3 min, 90%: 3 min, 85%: 3 min, 75%: 3 min). Next, the slides were rinsed in running water for 10 min and washed in distilled water for 5 min (repeated twice). Antigen retrieval was performed in citrate buffer (1.5 mg of sodium citrate and 0.2 mg of citric acid in 500 ml of distilled water) using a microwave, initially at medium–high power until boiling, then at low power for 15 min, and then allowed to cool naturally. After cooling, the slides were washed in running water for 10 min and in distilled water for 5 min (repeated twice). Subsequent treatment included 10 min with 3% hydrogen peroxide, followed by washing and blocking with 5% donkey serum at room temperature for 30–60 min. Primary antibodies were applied, and the samples were incubated overnight at 4 °C. After three washes with 10 × Tris-buffered saline (TBS), secondary antibodies were added, and the samples were incubated at 37 °C for 30 min. Following another three washes with 10 × TBS, TSA staining solution (Opal dye diluted 1:50) was applied. Antigen retrieval was repeated if needed, followed by staining with 4’,6’-diamidino-2-phenylindole (DAPI) for 3 min. Finally, a fluorescence anti-quenching reagent was used for mounting.

### Immunohistochemistry

The pretreatment for FFPE blocks for IBC and nIBC was consistent with the multiplex immunofluorescence procedure. After antigen retrieval, the slides were blocked with 5% donkey serum or 5% goat serum at room temperature for 30–60 min. Primary antibodies were applied, and the samples were incubated overnight at 4 °C. After three washes with 10 × TBS, secondary antibodies were added, and the samples were incubated at 37 °C for 30 min. The slides were then washed three times with 10 × TBS, followed by incubation with the diaminobenzidine (DAB) chromogen for 2 min. After being rinsed for 10 min under running water, the slides were washed twice with distilled water, stained with haematoxylin for 3 min, and then differentiated in a solution of 5 ml of concentrated hydrochloric acid and 500 ml of 75% ethanol three times. The slides were rinsed for 15 min until the water turned blue‒purple, followed by a graded ethanol series (70%: 10 s, 80%: 10 s, 90%: 10 s, 95%: 10 s, 100% × 2: 10 s), and then they were immersed in xylene for 10 s (repeated twice) before being mounted with neutral resin.

### Isolation of CD8 + T cells from PBMCs

Peripheral blood (10 mL) was collected from healthy donors into EDTA-coated tubes and processed within 2 h. PBMCs were isolated by Ficoll density gradient centrifugation (400 × g, 30 min, room temperature, no brake), followed by two washes with phosphate-buffered saline (PBS). CD8⁺ T cells were subsequently purified by negative selection using the CD8⁺ T Cell Isolation Kit (Miltenyi Biotec) according to the manufacturer’s protocol. Cell viability, determined by Trypan Blue exclusion, consistently exceeded 95%.

### Cytotoxicity assay of CD8 + T Cells

CD8⁺ T cells were isolated from healthy donor-derived PBMCs using magnetic bead-based negative selection (CD8⁺ Isolation Kit, Miltenyi Biotec), then activated with anti-CD3/CD28 antibodies (2 μg/mL, BioLegend) in the presence of interleukin-2 (IL-2, 50 IU/mL) for 48 h. Target cells included patient-derived primary IBC cells or tumor spheroids generated from co-culture systems. Effector CD8⁺ T cells were co-cultured with target cells at effector-to-target (E:T) ratios of 4:1 and 8:1 in 96-well plates for 4–24 h. Cytotoxicity was determined using a lactate dehydrogenase (LDH) release assay (Promega), with blank, spontaneous lysis, and Triton X-100-induced maximum lysis controls. Percent cytotoxicity was calculated according to the manufacturer’s instructions.

### Endothelial cell source

Human umbilical vein endothelial cells (HUVECs) were obtained from primary cultures and maintained in complete HUVEC medium (Meilunbio, Cat#PWL121) supplemented with 10% fetal bovine serum (FBS), 1% endothelial cell growth supplement, and 1% penicillin–streptomycin. Cells were incubated at 37 °C in a humidified atmosphere with 5% CO₂, and only those within six passages were used for subsequent experiments.

### Calcein-AM and ethidium homodimer-1 live/dead staining assay

HUVECs from control and experimental groups were incubated with 2 μM Calcein-AM and 4 μM Ethidium Homodimer-1 (EthD-1) (MCE, HY-D0093) for 10 min in the dark. Following PBS washes, cells were visualized using a fluorescence microscope or high-content imaging system. Viable cells exhibited green fluorescence (Calcein⁺), whereas dead cells showed red fluorescence (EthD-1⁺). The live/dead cell ratio was calculated to evaluate T cell-mediated or overall cytotoxicity.

### TNF neutralizing antibody and PTN neutralizing peptide

In B cell–endothelial cell co-cultures, anti-TNFα neutralizing antibody (Cell Signaling Technology, Cat#7321) and anti-PTN blocking peptide (MyBioSource, Cat#MBS427187) were added at a final concentration of 10 μg/mL. An equivalent concentration of isotype IgG served as the control. After 24 h of treatment, cytotoxicity, cell viability, and migratory capacity were evaluated.

### Isolation and culture of primary IBC cells

Fresh tumor tissues from inflammatory breast cancer (IBC) patients were collected within 2 h post-surgery under sterile conditions and immediately processed. After PBS washing, tissues were minced into ~ 1 mm^3^ fragments and enzymatically dissociated using the Primary Tumor Tissue Digestion Kit (Miltenyi Biotec, Cat#130–095-929) with the GentleMACS™ Tissue Dissociator, following the manufacturer’s instructions. The resulting suspension was filtered through a 70 μm cell strainer to obtain single cells. Cells were cultured in RPMI-1640 medium (Gibco) supplemented with 10% fetal bovine serum (FBS, Gibco) and 1% penicillin–streptomycin at 37 °C in 5% CO₂. Once adherent, media were refreshed every 2–3 days, and passaging was performed based on confluence. Cell identity was confirmed by immunofluorescence or flow cytometry, with EpCAM⁺/CK19⁺/CD45⁻ profiles to ensure epithelial origin and exclude immune cell contamination.

### IBC transmigration assay

Endothelial cells were pretreated for 24 h with B cell-derived supernatant supplemented with recombinant PTN (Proteintech, Cat#HZ-1278). Cells were then digested and seeded into the upper chamber of a Transwell system (Corning, Cat#3422) pre-coated with HUVECs to establish an endothelial barrier. Calcein-labeled IBC cells (1 × 10^5^/well) were added to the upper chamber, and complete culture medium was placed in the lower chamber. After 48 h of co-culture, non-migrated cells were removed from the upper chamber using a cotton swab. Migrated calcein-positive cells in the lower chamber were fixed and counted under a fluorescence microscope. Images from five randomly selected fields per well were analyzed to calculate the average number of migrating cells. In some experiments, neutralizing antibodies against TNFα or PTN were included as intervention controls.

### Isolation of macrophages and induction of M0 differentiation from PBMCs

PBMCs were isolated by Ficoll density gradient centrifugation and plated in serum-free dishes. After 2 h, non-adherent cells were removed, and adherent mononuclear cells were cultured in RPMI-1640 medium supplemented with M-CSF (PeproTech, Cat#300–35-50UG). After 7 days of induction, cells differentiated into M0 macrophages, as confirmed by CD14⁺/CD68⁺ immunostaining.

### Isolation of B cells from PBMCs

PBMCs were isolated from healthy donor peripheral blood by Ficoll-Paque density gradient centrifugation. B cells were positively selected using the B Cell Isolation Kit (Miltenyi Biotec, Cat#130–091-151) with anti-CD19 magnetic antibodies, and separated using a MACS sorting column. The isolated B cells were cultured in RPMI-1640 medium supplemented with 10% fetal bovine serum (FBS) and 1% penicillin–streptomycin at 37 °C in a 5% CO₂ atmosphere.

### In vitro HUVEC tube formation assay

Fifty microliters of Matrigel were added to each well of a 96-well plate and incubated at 37 °C for 1 h to solidify. A 50 µL cell suspension was then seeded onto the Matrigel-coated wells. After 24 h of incubation, photomicrographs were taken using light microscopy. The total tube area was quantified by measuring the mean pixel density from five randomly selected microscopic fields using ImageJ software (http://rsb.info.nih.gov/nih-image/).

### NCL siRNA transfection of B cells

Human B lymphocytes were transfected with NCL siRNA (50 nM, GenePharma) using Lipofectamine RNAiMAX (Thermo Fisher Scientific) in serum-free Opti-MEM medium, following the manufacturer’s instructions. The siRNA-Lipofectamine mixture was incubated for 10 min at room temperature and then added to logarithmically growing B cells (1 × 10⁶ cells/well). After 48 h of culture in RPMI-1640 medium supplemented with 10% fetal bovine serum, transfection efficiency was assessed using fluorescently labeled siRNA or quantitative real-time PCR (qRT-PCR).

### Establishment of stable RIPK3 knockdown in endothelial cells via lentiviral infection

Human umbilical vein endothelial cells (HUVECs) were transduced with lentiviral particles to achieve stable knockdown of RIPK3. Lentiviruses encoding RIPK3-targeting shRNA (GeneChem) were produced by co-transfecting the shRNA plasmid and packaging plasmids into HEK293T cells. The viral supernatants were harvested, concentrated, and used to infect HUVECs at 50% confluence in 6-well plates in the presence of 8 μg/mL Polybrene for 24 h. After replacing the medium with fresh culture medium, cells were maintained for 48–72 h. RIPK3 knockdown efficiency was verified by quantitative real-time PCR (qRT-PCR) and Western blotting.

### Chromatin immunoprecipitation (ChIP)

Cells were cross-linked with 1% formaldehyde for 10 min, quenched, and lysed. Chromatin was sheared by sonication to achieve DNA fragments of 200–500 bp. Chromatin immunoprecipitation (ChIP) assays were performed using a commercial kit (Millipore, Cat#17–295) according to the manufacturer’s instructions. Antibodies against NFAT1, ATF2, and c-Jun were incubated with the chromatin overnight, followed by enrichment with Protein A/G magnetic beads. After washing and reverse cross-linking, DNA was purified and analyzed by quantitative PCR to assess NFAT1 binding to the LAG3 and TIGIT promoters, and ATF2 and c-Jun binding to the TNF promoter. Normal IgG was used as a negative control.

### Statistical analysis

Statistical analyses were conducted using R software (version 4.1.3, https://www.r-project.org/). Significance was determined with *P* values less than 0.05 from two-sided Wilcoxon tests and t tests. Data visualization and statistical analysis were performed using GraphPad Prism 9 (GraphPad Software).

## Results

### Lymphocyte infiltration in the HER2 + IBC TME exceeds that in nIBC

**Fig. 1:**
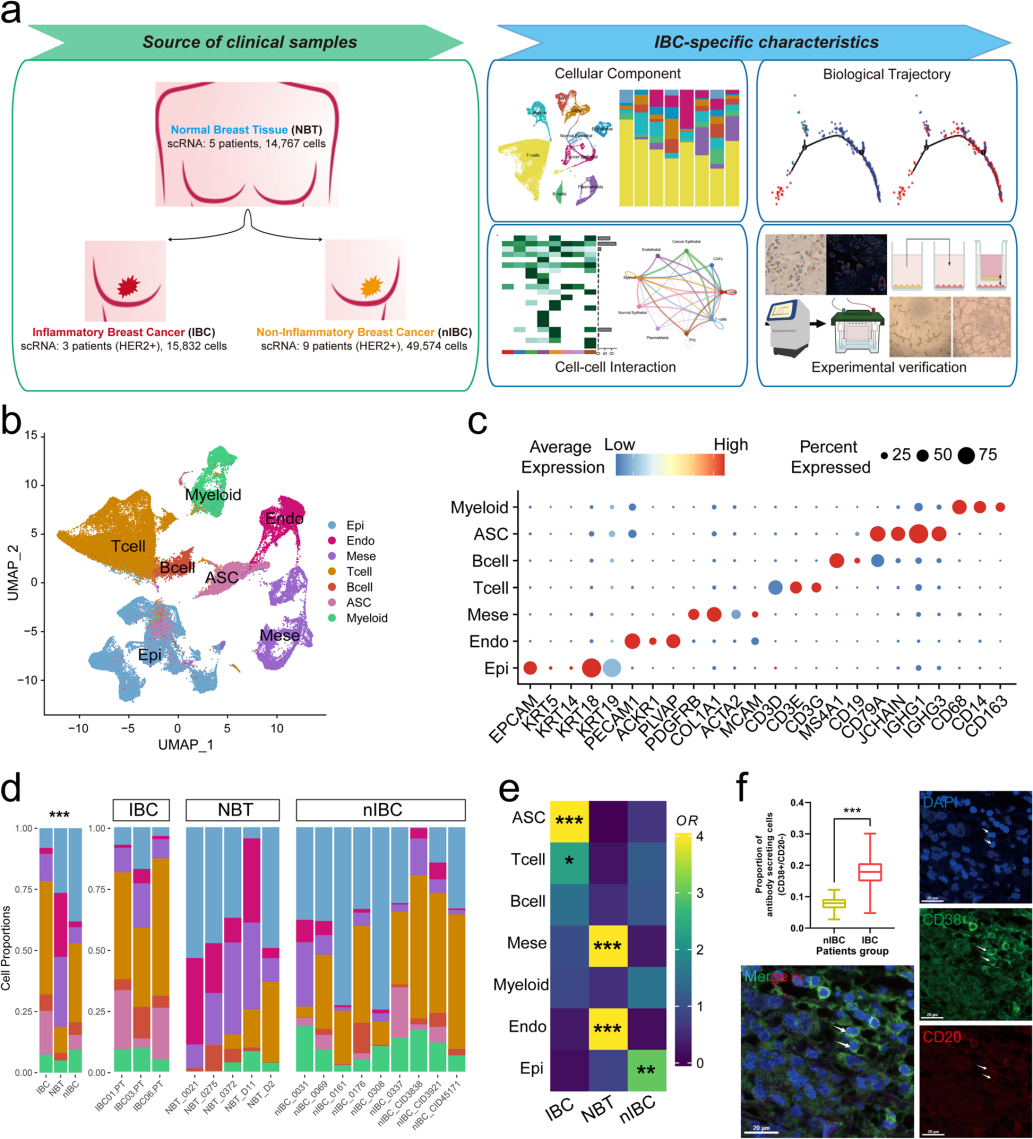
Single-cell transcriptome landscape of HER2 + inflammatory breast cancer patients. **a** Flowchart of dataset construction and analysis for this study. **b** UMAP plots of major cell type annotations. **c** Demonstration of differences in marker gene expression between different cell types using bubble plots. **d** Utilising bar graphs to demonstrate the proportional distribution of major cell types between different patients. **e** Heatmaps illustrating the odds ratios (ORs) of various cell types across different tissue sources. **f** Comparison of the proportion of antibody-secreting cells infiltrating in patients with IBC and nIBC using multiplex immunofluorescence

The HER2 + molecular subtype is the most common subtype among IBC patients and is more prevalent than the corresponding subtype in nIBC (nIBC), yet the efficacy of treatment for IBC remains considerably lower [6]. To explore the TME of IBC patients and clarify the changes in their cellular components and molecular characteristics, we integrated single-cell data from three sources of breast tissues, including 3 patients with HER2 + IBC (self-assessed data from this study), 9 patients with HER2 + nIBC (GSE176078 and GSE161529), and 5 NBT (normal breast tissues, GSE161529 and GSE164898). We controlled for confounding variables by selecting patients within the same age range, with the clinical details provided in Supplementary Table S1. The analysis framework is shown in Fig. 1a, which shows minimal batch effects (Extended Data Fig. S1ab) and a clear spatial distribution of classical marker genes (Extended Data Fig. S1c). Through unsupervised clustering and marker gene expression analysis [36], we identified various cell types in breast tissue, including epithelial cells (EPCAM, KRT18), endothelial cells (PECAM1, PLVAP), stromal cells (PDGFRB, COL1A1), T lymphocytes (CD3D, CD3E), B lymphocytes (MS4A1, CD79A), antibody-secreting cells (JCHAIN, IGHG1), and myeloid cells (CD68, CD14) (Fig. 1b). The distinct expression of classical markers validated the accuracy of the cell type annotations (Fig. 1c).

A comparison of cell components across tissue types revealed that normal breast tissues predominantly consisted of epithelial, endothelial, and stromal cells, whereas tumour tissues (IBC and nIBC) presented increased immune cell infiltration (Fig. 1d). Endothelial and stromal cells were significantly reduced in tumours compared with normal tissues, with no significant difference between IBC and nIBC (Fig. 1e). Immune cells, including T lymphocytes, antibody-secreting cells, and myeloid cells, are markedly elevated in tumours, with higher lymphocyte infiltration in IBC than nIBC (Fig. 1e). Notably, antibody-secreting cells were significantly enriched in IBC and nearly absent in normal breast tissue (Fig. 1e). Immunofluorescence of IBC and nIBC tissue samples confirmed the high proportion of antibody-secreting cells in IBC tumours (Fig. 1f). These findings highlight a greater level of lymphocyte infiltration, especially of antibody-secreting cells, in IBC tumours.

### T cells in the HER2 + IBC tumour microenvironment exhibit a dual phenotype of high activation and high exhaustion

**Fig. 2:**
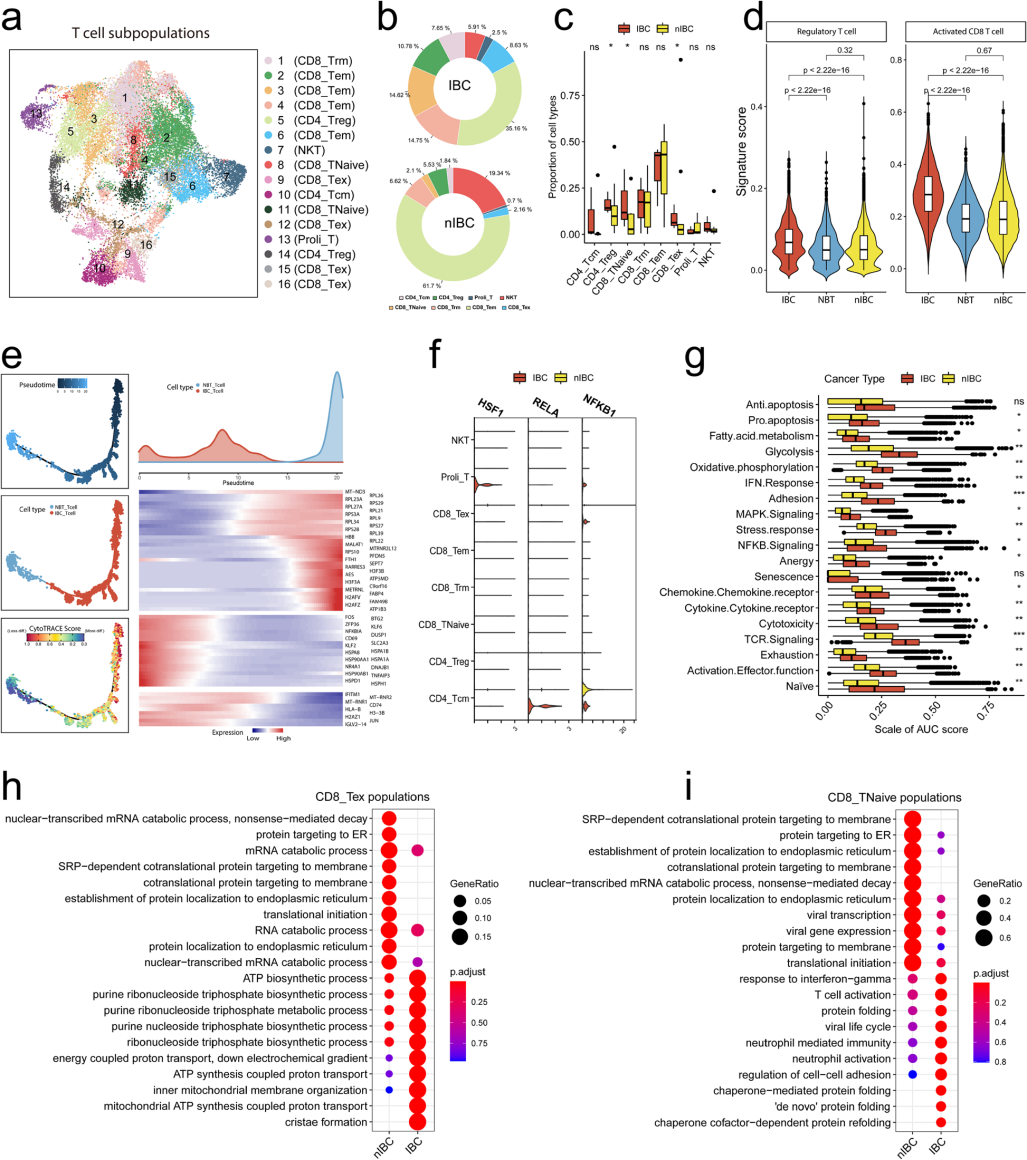
Heterogeneity of tumour-infiltrating T cells. **a** Demonstration of T cell subpopulations and corresponding annotations using UMAP plots. **b-c** Demonstration of the percentage of various T cell subpopulations in IBCs and nIBCs using circle plots (**b**), and presentation of the results of statistical tests using box-and-line plots (**c**). **d** Demonstration of the differences in the two T-cell-associated signature gene set scores between sample types using violin plots. **e** Proposed time-series analysis to demonstrate the dynamic plasticity of T cells in IBC. **f** Demonstrating the expression of three transcription factors in various T cell subpopulations using violin plots. **g** Comparison of the differences in the 19 T-cell functional state-related gene set scores between IBC and nIBC using box-and-line plots. **h-i** Demonstration of GOBP enrichment of differentially expressed genes between IBC and nIBC using bubble plots for CD8_TNaive (**h**) and CD8_Tex (**i**) cells, respectively

Tumour-infiltrating T cells have diverse functional states and play important roles in tumour progression and treatment tolerance. To compare IBC and nIBC tumour-infiltrating T lymphocytes in terms of cell state and function, we reclustered all T lymphocytes into 16 subpopulations and defined eight major T lymphocyte states using relevant molecular markers (Fig. 2a, Extended Data Fig. S2a, b), including CD4 + central memory T cells (CD4_Tcm: C10), CD4 + regulatory T cells (CD4_Treg: C5, C14), CD8 + naive T cells (CD8_TNaive: C8, C11), CD8 + tissue-resident memory T cells (CD8_Trm: C1), CD8 + effector memory T cells (CD8_Tem: C2, C3, C4, C6), CD8 + exhausted T cells (CD8_Tex: C9, C12, C15, C16), proliferating T cells (Proli_T: C13), and natural killer T cells (NKT: C7) (Fig. 2a). We subsequently compared the percentages of T-cell status in IBC and nIBC tumours and found that the T-cell types with the highest percentages in IBC tumours were CD8_Tem (approximately 35.16%), CD8_Trm (approximately 14.75%), and CD8_TNaive (approximately 14.62%) (Fig. 2b). The T-cell types with significant differences were CD8_TNaive (IBC 14.62% vs. nIBC 2.1%), CD4_Treg (IBC 10.78% vs. nIBC 5.53%) and CD8_Tex (IBC 8.63% vs. nIBC 2.16%) (Fig. 2c). When all T cells were scored on the basis of the set of genes related to T-cell functional status, we found that IBC tumour-infiltrating T cells presented increased levels of regulatory and CD8 + activated T cells (Fig. 2d, Extended Data Fig. S2e). By comparing the differences in T-cell activation and differentiation between IBC and nIBC tumours by pseudotime analysis, we found that the density distribution of IBC tumour-infiltrating T cells was more dispersed than that in normal breast tissues, whereas the density distribution of nIBC tumour-infiltrating T cells overlapped more with that in normal breast tissues, suggesting that the overall difference between IBC tumour-infiltrating T cells and normal breast tissue-infiltrating T cells was more significant (Extended Data Fig. S2d). Furthermore, we used correlation analysis to identify gene modules associated with pseudotime in IBC and found that the IBC direction had high signals of T-cell activation molecules (CD69, FOS) and heat shock protein molecules (HSPA1A, HSPA1B, HSPA8) (Fig. 2e). Transcription factor enrichment analysis was performed for genes with high signalling in the specific direction of IBC, and we found that these genes were regulated by three main inflammation-associated transcription factors (HSF1, RELA, and NFKB1), of which HSF1 was highly expressed mainly in Proli_T of IBC, RELA was highly expressed mainly in CD4_Tcm of IBC, and NFKB1 was highly expressed mainly in CD4_Tcm of IBC and CD8_Tex (Fig. 2f).

By scoring all the T cells on the basis of the T-cell functional response-related gene sets, we found that the enriched signals of all the gene sets had a more active status in IBC, with adhesion and TCR signalling having the most significant differences (Fig. 2g), indicating a more active T-cell-mediated immune response in HER2 + IBC. Given that the proportions of CD8_TNaive and CD8_Tex cells were significantly greater in IBC than in nIBC, we performed differential gene enrichment analyses of these two cell subpopulations for IBC compared with nIBC and found that CD8_TNaive cells in IBC tumours presented greater levels of activation (response to interferon gamma, T-cell activation, and neutrophil-mediated immunity) and a more pronounced protein folding response (chaperone-mediated protein folding, de novo protein folding) in IBC tumours, suggesting that protein synthesis in T cells is enhanced to mediate a stronger T-cell immune response (Fig. 2h). Moreover, we found that CD8_ Tex cells had stronger mitochondrial energy metabolism-related signals (ATP biosynthetic process, purine ribonucleoside triphosphate biosynthetic process, and crista formation) in IBC tumours (Fig. 2i). These results suggest that IBC tumour-infiltrating T cells have large differences in terms of cellular function and status compared with nIBC cells, which is mainly reflected in the fact that IBC tumour-infiltrating T cells have a more pronounced CD8 + T-cell immune response but also show greater activation of signals from immunosuppressive states, such as the CD4_Treg and CD8_Tex cell statuses. Thus, the highly activated and highly suppressive molecular phenotype of T cells is an important manifestation of the ability of IBC tumour cells to evade immune surveillance.

### B cells in the HER2 + IBC tumour microenvironment exhibit a preference for extrafollicular developmental pathways

**Fig. 3:**
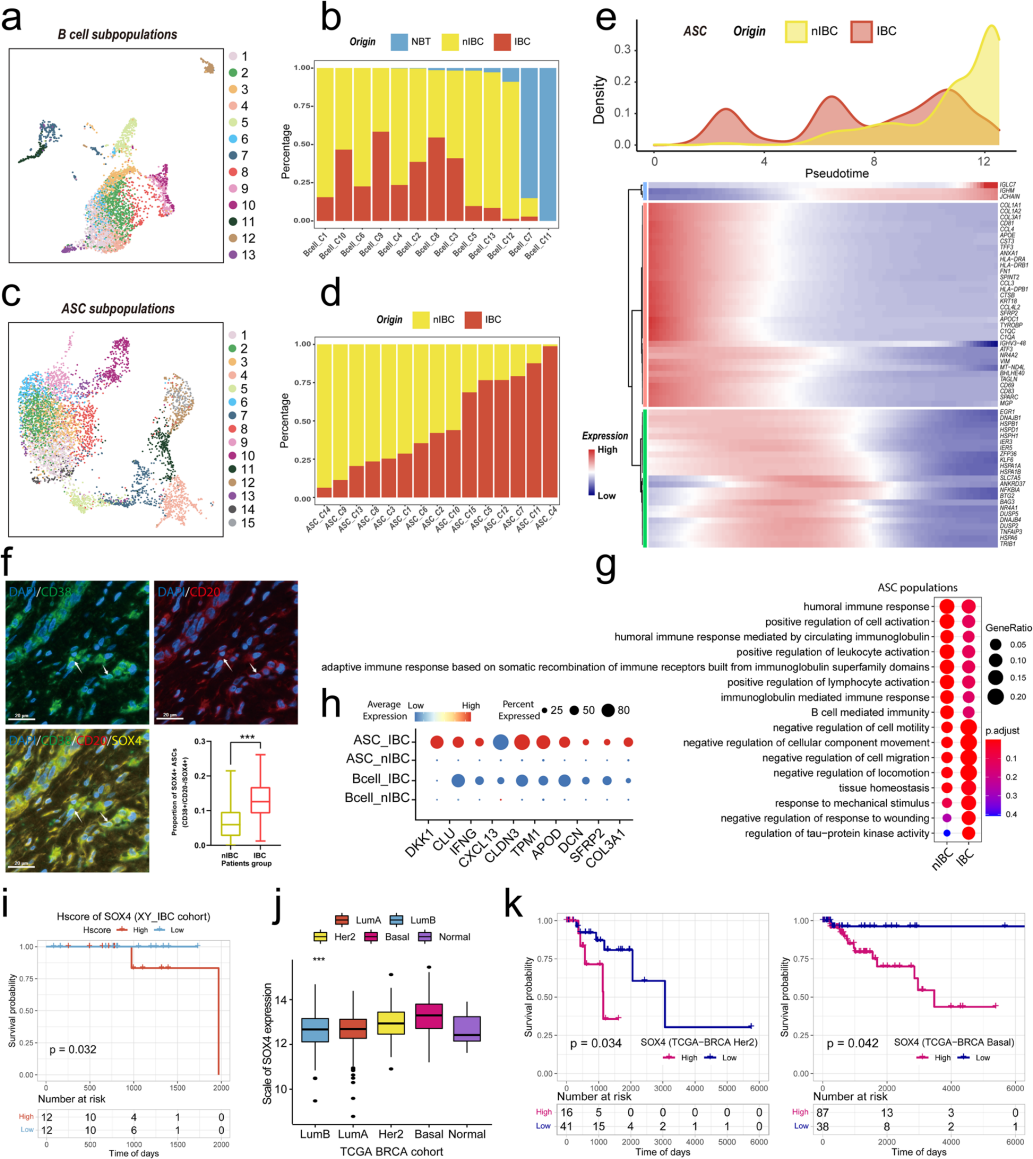
Heterogeneity of tumour-infiltrating B cells and antibody-secreting cells. **a** Demonstration of the distribution of B cell subpopulations using UMAP plots. **b** Demonstrates the proportional distribution of tissue types in different B cell subpopulations using bar graphs. **c** Demonstrate the distribution of antibody-secreting cell subpopulations using UMAP plots. **d** Demonstrate the proportional distribution of tissue types in different antibody-secreting cell subpopulations using a bar chart. **e** Proposed time series analysis to demonstrate the dynamic plasticity of antibody-secreting cells in IBC. **f** Comparison of the proportion of SOX4 + antibody-secreting cells infiltrating in IBC and nIBC patients using multiplex immunofluorescence. **g** Demonstration of GOBP enrichment of differentially expressed genes in antibody-secreting cells between IBC and nIBC using bubble plots. **h** Demonstration of 10 genes specifically highly expressed in B cells and antibody-secreting cells in IBC using bubble plots. **i** Survival curve analysis (Kaplan–Meier method) demonstrated the prognostic significance of SOX4 gene expression in the HER2 + IBC cohort (log-rank *p* = 0.032). **j-k** Demonstration of differential expression (**i**) and prognostic significance (**j**) of SOX4 genes in different molecular subtypes of the TCGA breast cancer cohort using box-and-line plots and survival curves, respectively

Tumour-infiltrating B cells also play a very important role in the regulation of the tumour immune microenvironment. To compare the differences in the activation and differentiation of B lymphocytes in IBC and nIBC tumours, we used unsupervised clustering analysis to subdivide B cells and antibody-secreting cells into subpopulations. First, we reclustered B cells into 13 subpopulations, which were found to be more clustered among B-cell subpopulations from UMAP plots (Fig. 3a). A statistical comparison of the proportions of B-cell subpopulations of different tissue origins revealed a large difference in the distributions of B-cell subpopulations in tumour tissues versus normal tissues (predominantly C7 and C11), whereas no B-cell subpopulations were particularly enriched in IBC tumours (Fig. 3b). Our differential enrichment analysis of all B cells in IBC and nIBC revealed that IBC tumour-infiltrating B cells have a high level of immunoreactive signals, including myeloid dendritic cell activation, positive regulation of monooxygenase activity, and major histocompatibility complex (MHC) class II biosynthetic processes (Extended Data Fig. S3a), in which IFNG and GNLY were significantly overexpressed in B cells of IBC (Extended Data Fig. S3b).

Subsequently, we reclustered the antibody-secreting cells into 15 subpopulations, which were found to be more dispersed among the antibody-secreting cell subpopulations from the UMAP plots (Fig. 3c). A statistical comparison of the proportion of the tissue origin of the antibody-secreting cell subpopulations revealed that the distribution of the antibody-secreting cell subpopulations enriched in IBC and nIBC tumours had a significant gradient change, suggesting that there was a process of differential differentiation of the various subpopulations of the antibody-secreting cells between IBC and nIBC (Fig. 3d). We subsequently performed a pseudotime analysis of all antibody-secreting cells from IBC and nIBC tumours and found that IBC tumour-infiltrating antibody-secreting cells had a more activated differentiation state, with antibody-secreting cells early in the pseudotime direction of IBC differentiation expressing high levels of the B-cell activation molecules CD69 and CD83 (Fig. 3e). In addition, we found that antibody-secreting cells from IBC tumours underexpressed IGHM compared with those from nIBC tumours, suggesting a more pronounced antibody class switch in antibody-secreting cells (Fig. 3e, Extended Data Fig. S3d). In terms of immune bank (VDJ) gene expression in B-cell receptor (BCR), we found that antibody-secreting cells highly expressed IGHV3-48 (Fig. 3e). In addition, IBC-infiltrating antibody-secreting cells highly expressed several molecular markers of antigen presentation, HLA-DRA and HLA-DRB1, compared to nIBC-infiltrating antibody-secreting cells (Fig. 3e), suggesting that IBC-infiltrating antibody-secreting cells have a certain antigen-presenting ability. Notably, IBC-infiltrating antibody-secreting cells specifically expressed several collagen-associated molecules, such as COL1A1, COL1A2 and COL3A1 (Fig. 3e, h). According to the literature, collagen-like proteins may promote the activation of fibroblasts in certain tumours [37]; thus, IBC-infiltrating ASCs may contribute to tumour progression through the activation of cancer-associated fibroblasts (CAFs).

Furthermore, we performed gene expression analysis on the basis of markers of B-cell activation and immaturity and found that IBC tumour-infiltrating B cells presented high levels of activation signals, with three molecules, CD19, BLK, and CD79B, being expressed at significantly higher levels in IBC tumour-infiltrating B cells than in nIBC (Extended Data Fig. S3c). Moreover, we found that IBC tumour-infiltrating B cells also presented high levels of immature signals, which was reflected mainly in the significantly high expression of five molecules, CD22, FCRL1, FCRLA, HVCN1, and SP100, in IBC tumour-infiltrating B cells (Extended Data Fig. S3c). We also found a number of molecules with high expression of both activation and immaturity signals in antibody-secreting cells, including widely expressed activation signalling molecules such as CD38, CD27, and TNFRSF17, and widely expressed immature signalling molecules such as TXNIP and FCRL5, but the expression of these molecules did not differ significantly between IBC and nIBC samples (Extended Data Fig. S3c). The above results suggest that IBC-infiltrating B cells need to enter a highly activated state first during differentiation, which promotes the differentiation of B cells into antibody-secreting cells. We subsequently performed an overall differential gene expression analysis of antibody-secreting cells from IBC and nIBC, and found that the antibody-secreting cell subpopulation (C4, C11) enriched in IBC tumours specifically expressed high levels of the SOX4 gene (Extended Data Fig. S3d) and that the antibody-secreting cell subpopulation C4 exhibited broad expression of a wide range of molecular markers of immune cells (Extended Data Fig S3e). Through differential gene enrichment analysis, we found that IBC tumour-infiltrating antibody-secreting cells presented highly enriched signals related mainly to cell adhesion and cell motility, of which the pathways related to cell adhesion were the regulation of tau-protein kinase activity, negative regulation of the response to wounding, response to mechanical stress, and negative regulation of cell motility, and those related to cell adhesion were the response to wounding, response to mechanical stimulus, negative regulation of locomotion, negative regulation of cell migration, negative regulation of cellular component movement, and negative regulation of cell motility (Fig. 3g). On the basis of the pathways specifically enriched in antibody-secreting cells infiltrated by IBC tumours, we found that the relevant molecules involved in these pathways (DKK1, CLU, IFNG, CXCL13, CLDN3, TPM1, APOD, DCN, SFRP2, and COL3A1) had a broadly high expression profile not only in antibody-secreting cells but also in B cells (Fig. 3h).

Furthermore, using paraffin samples from patients with IBC and nIBC, we demonstrated by multiplex immunofluorescence that the degree of infiltration of SOX4 + antibody-secreting cells was significantly greater in IBC than in nIBC (Fig. 3f). Moreover, we compared the differences in the expression of SOX4 molecules among the four molecular subtypes in the TCGA-BRCA dataset and found that the expression of SOX4 was significantly greater in tumours than in normal tissues, with higher expression levels in the basal and HER2 subtypes (Fig. 3j), and its expression level had better prognostic stratification significance in the basal and HER2 subtypes (Fig. 3k). We compared the differences in SOX4 expression among the 33 cancers in TCGA and found that its expression level was significantly greater than that in normal tissues in most cancer types (Extended Data Fig S4a), but there was greater heterogeneity in its prognostic significance (Extended Data Fig S4b-e). In the HER2 + IBC cohort, patients with higher expression of SOX4 showed a poorer prognosis (Fig. 3i). Taken together, the highly activated and immature molecular phenotype of HER2 + IBC tumour-infiltrating B cells and antibody-secreting cells reflects the bias of B cells towards extrafollicular development into antibody-secreting cells. Additionally, the SOX4 molecule is an important marker for IBC tumour-infiltrating antibody-secreting cells, which may mediate the high expression of collagen-related molecules by IBC tumour-infiltrating antibody-secreting cells to promote CAF proliferation.

### Myeloid cells and fibroblasts in the HER2 + IBC tumour microenvironment have a greater inhibitory effect

**Fig. 4:**
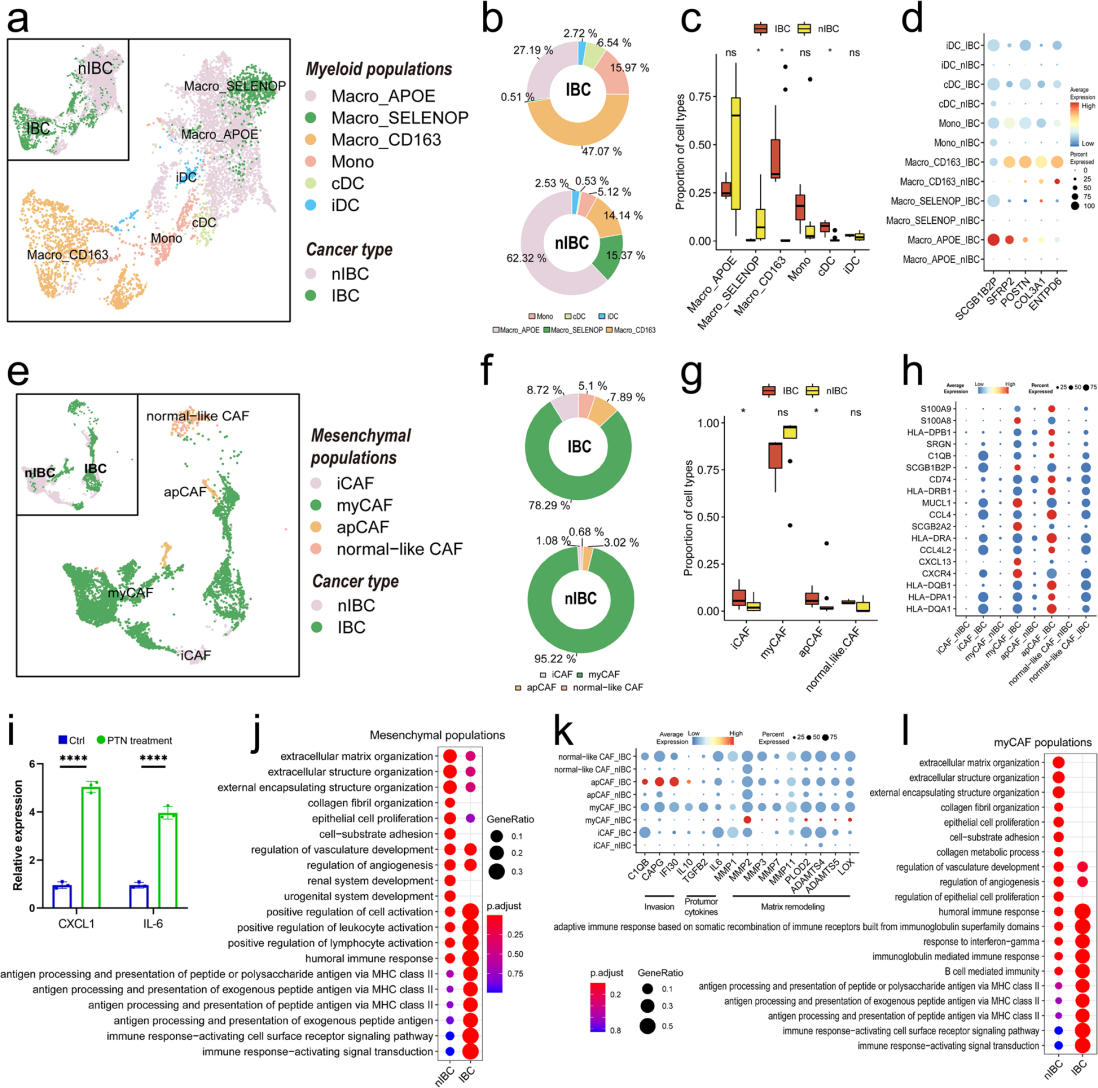
Heterogeneity of tumour-infiltrating myeloid and mesenchymal cells. **a** Demonstration of the distribution of myeloid cell subpopulations using UMAP plots. **b-c** Demonstration of the percentage of various myeloid cell subpopulations in IBCs and nIBCs using circle plots (**b**), and demonstration of the results of statistical tests using box-and-line plots (**c**). **d** Demonstration of five genes with high myeloid cell-specific expression in IBC using bubble plots. **e** Demonstration of the distribution of myeloid cell subpopulations using UMAP plots. **f-g** Demonstration of the percentage of various stromal cell subpopulations in IBC and nIBC using circle graphs (**f**) and presentation of the results of the statistical tests using box-and-line plots (**g**). **h** Demonstration of 18 genes with high stromal cell-specific expression in IBC using bubble plots. **i** Bar chart analysis illustrated the PTN-induced modulation of iCAF-associated molecular markers (CXCL1 and IL-6) in CAFs, with quantitative expression data derived from three independent experiments (one-way ANOVA, *** indicates that p < 0.001). **j** Demonstrates GOBP enrichment of differentially expressed genes between IBC and nIBC in stromal cells using bubble plots. **k** Demonstrates the expression of pro-tumour-associated molecules in stromal cells using bubble plots. **l** Demonstration of GOBP enrichment of myCAF in differentially expressed genes between IBC and nIBC using bubble plots

To compare the functional status and gene expression heterogeneity of myeloid cells in IBC and nIBC tumours, we integrated unsupervised clustering analysis and classical molecular marker expression analysis of myeloid cells to subdivide myeloid cells into subpopulations. First, we reclustered myeloid cells into 18 subpopulations (Extended Data Fig. S5ab) and found that myeloid cell subpopulations differed greatly from each other in the UMAP plot and that the subpopulation distribution had some tissue origin specificity (Fig. 4a). On the basis of the expression of classical molecular markers of myeloid cells (Extended Data Fig. S5a), we classified all myeloid cells into six major functional cell states, namely, APOE + macrophages (Macro_APOE: C1, C2, C4, C5, C11, C15, C18), SELENOP + macrophages (Macro_SELENOP: C3), CD163 + macrophages (Macro_CD163: C6, C8, C10, C12, C14, C16), monocytes (Mono: C7, C9), classical dendritic cells (cDC: C13), and inflammatory dendritic cells (iDC: C17) (Fig. 4a). Statistics on the percentage distribution of myeloid cell functional status revealed that myeloid cells in IBC tumours were dominated by CD163 + macrophages (approximately 47.07%), APOE + macrophages (approximately 27.19%), and monocytes (approximately 15.97%) as the major cellular components, whereas myeloid cells in nIBC tumours were dominated by APOE + macrophages (approximately 62.32%), SELENOP + macrophages (approximately 15.37%), and CD163 + macrophages (approximately 14.14%) as the main cellular components (Fig. 4b), whereas CD163 + macrophages were significantly enriched in IBC tumours (IBC 47.07% vs. nIBC 14.14%) (Fig. 4c). Moreover, we identified five molecules (SCGB1B2P, SFRP2, POSTN, COL3A1, ENTPD6) that were universally overexpressed in medullary cells infiltrated by IBC tumours (Fig. 4d). A strong correlation between myeloid cells in the direction of IBC differentiation and high expression of the transcription factor SPP1 was found by pseudotime analysis (Extended Data Fig. S5d). Moreover, the antigen-presenting genes HLA-DQA1 and HLA-DQB1 were strongly correlated with pseudotime in IBC tumour-infiltrating myeloid cells (Extended Data Fig. S5d), suggesting that IBC tumour-infiltrating myeloid cells may promote humoral immunity by enhancing their antigen-presenting ability. Furthermore, we found that tumour-infiltrating myeloid cells had high expression levels of tumour-associated macrophage markers and monocyte-derived suppressor cell markers by gene set enrichment analysis (Extended Data Fig. S5e). These results suggest that tumour-infiltrating myeloid cells as a whole exhibit a greater degree of suppression and that IBC tumour-infiltrating myeloid cells are predominantly CD163 + macrophages, whereas myeloid cells in nIBC tumours are predominantly primary APOE + macrophages, suggesting that IBC tumour-infiltrating myeloid cells have a greater state of immunosuppressive properties.

**Fig. 5:**
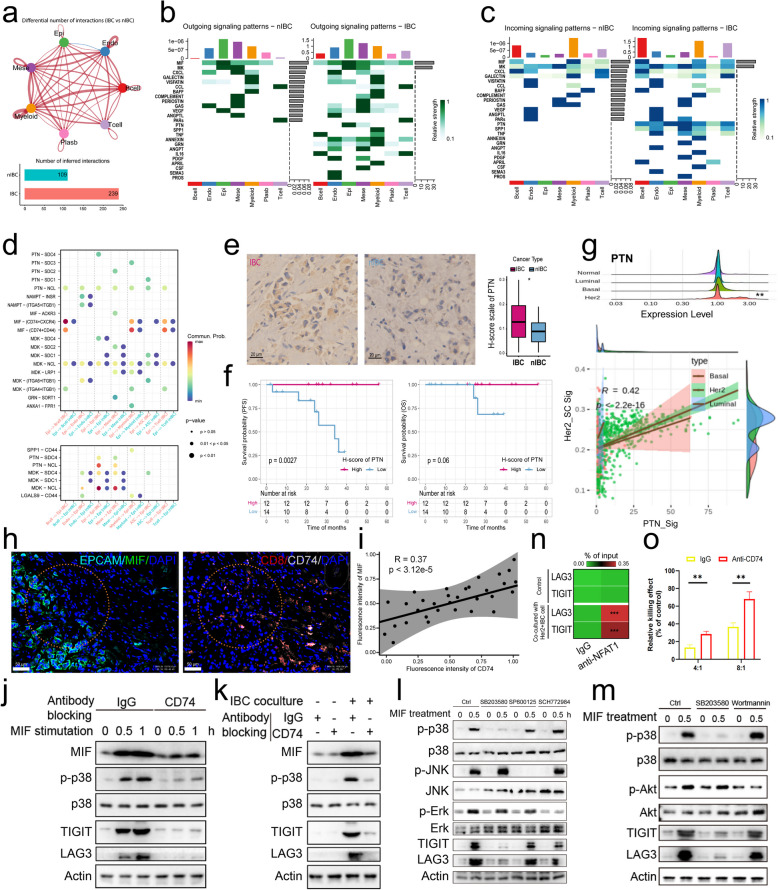
Comparison of cellular communication in the tumour microenvironment. **a** Quantitative analysis of intercellular crosstalk heterogeneity among major cell types revealed differential interaction profiles. The force-directed network visualisation encoded interaction strength through edge weight, while the accompanying bar plot quantified ligand-receptor pairs. **b-c** Use of heatmaps to demonstrate the variation in the strength of output signals (**b**) and input signals (**c**) in the major cell types, respectively. **d** Demonstrate the strength of ligand gene pairs with significant differences in cellular interactions using bubble plots. **e** Differences in the expression of PTN molecules in tumour tissues from IBC and nIBC patients were compared using immunohistochemistry. **f** The relationship between the expression of PTN molecules and progression-free survival (left panel) and overall survival (right panel) of IBC patients was demonstrated using survival curves. **g** The expression differences (above panel) of PTN molecules among different molecular subtypes of IBC and their correlations (below panel) with subtype characteristic scores. **h-i** Display the co-localization between MIF and CD74 based on multicolor fluorescence. **j** IgG or CD74 neutralizing antibodies were incubated in the culture medium of CD8 + T cells, followed by co-culture with IBC tumor cells for different time points. Western blotting was performed to analyze the expression of indicated proteins in CD8 + T cells. **k** CD8 + T cells were pretreated with p38, JNK, or Erk inhibitors for 30 min, followed by stimulation with recombinant MIF protein for varying durations. Western blotting was used to detect the expression of indicated proteins in T cells. **l** CD8 + T cells were pretreated with p38 or Akt inhibitors for 30 min, followed by stimulation with recombinant MIF protein for varying time points. Western blotting was used to detect the expression of indicated proteins in T cells. **m** Following either normal culture or co-culture with HER2 + IBC cells for 24 h, ChIP was performed to detect the binding of NFAT1 to the LAG3/TIGIT motif in the TNF promoter of CD8 + T cells. **n** IgG or CD74 neutralizing antibodies were incubated in the culture medium, followed by stimulation of CD8 + T cells with recombinant MIF protein for varying durations. Western blotting was used to detect the expression of indicated proteins in T cells. **o** CD8 + T cells were co-cultured with HER2 + IBC cells in the presence of either IgG or CD74 neutralizing antibodies. Cellular cytotoxicity mediated by CD8 + T cells was assessed

To compare the functional status and molecular heterogeneity of IBC and nIBC tumour-infiltrating mesenchymal cells, we subdivided the mesenchymal cells into subpopulations using the same method described above. First, we reclustered the mesenchymal cells into 21 subpopulations (Extended Data Fig. S6a, b), and the UMAP plot revealed that the mesenchymal cell subpopulations were relatively dispersed from each other and that the subpopulation distributions had some tissue origin specificity (Fig. 4e). We subsequently classified tumour-infiltrating mesenchymal stromal cells into four main functional transitions on the basis of classical molecular markers (Extended Data Fig. S6a): inflammatory CAFs (iCAFs: C1, C3, C4, C9, C13, and C16), myofibroblast-like CAFs (myCAFs: C6, C7, C8, C10, C12, C14, C17, and C19), antigen-presenting CAFs (apCAFs: C20 and C21), and normal-like fibroblasts (NFs: C2, C5, and C15) (Fig. 4e). Counting the proportion of distribution of mesenchymal cell functional status, we found that the highest percentage of abundance of both IBC and nIBC tumour-infiltrating mesenchymal cells was in myofibroblastic-like CAFs (IBC: 78%, nIBC: 95%) (Fig. 4f), and notably, the percentages of IBC tumour-infiltrating inflammatory CAFs (IBC 8.72% vs. nIBC 1.08%) and antigen-presenting CAFs (IBC 7.89% vs. nIBC 3.02%) were both significantly greater than those of nIBC (Fig. 4g). Moreover, we found that inflammation-associated molecules (CXCR4, CCL4, CCL4L2, and CD74) and antigen-presentation-associated molecules (HLA-DQA1, HLA-DPA1, and HLA-DRA) had relatively widespread high expression levels in all mesenchymal cells of IBC tumours (Fig. 4h), and the immune-responsive MHC class II antigen-presentation molecule CD74 in IBC tumours infiltrating apCAFs had significantly high expression levels (Fig. 4h). By pseudotime analysis, mesenchymal stromal cells in the direction of IBC differentiation were found to be strongly correlated with high expression of the growth factor IGFBP7 (Extended Data Fig. S6d). Our differential enrichment analysis of all mesenchymal cells infiltrated by IBC and nIBC tumours revealed that the signalling pathways that were highly enriched in IBC tumour-infiltrating mesenchymal cells were associated mainly with immune cell activation, exogenous antigen presentation, and angiogenesis (Fig. 4j). By scoring the tumour microenvironment-related gene sets, we found that tumour-infiltrating mesenchymal cells expressed many stromal remodelling- and invasiveness-related molecules (Extended Data Fig. S6e), of which the invasiveness-related molecules C1QB, CAPG, and IFI30 were highly expressed specifically in the IBC tumour-infiltrating mesenchymal cells (Fig. 4k). Further differential enrichment analysis of all myCAFs in IBC and nIBC tumours revealed that the signalling pathways with high enrichment of myCAFs in IBC tumour infiltrates were also associated mainly with immune cell activation, exogenous antigen presentation, and angiogenesis (Fig. 4l). The results of cell communication analysis suggest that tumour cells can establish an interaction relationship with CAF by expressing PTN (Fig. 5b, c). The PTN receptor nucleolin (NCL) is highly expressed in stromal cells (Extended Data Fig. 7a) and exhibits a significant positive correlation with CAF signatures within this population (Extended Data Fig. 7b). Building on our recent work demonstrating that PTN drives myCAF-to-iCAF transition in colorectal cancer [38], we hypothesise that PTN similarly promotes myCAF-to-iCAF reprogramming in HER2 + IBC, potentially contributing to its aggressive phenotype. To confirm the role of PTN in the remodeling of CAF subtypes, we stimulated primary CAF cells isolated from HER2 + IBC with PTN. The results showed that PTN could significantly promote the expression of iCAF markers CXCL1 and IL-6 in CAF cells (Fig. 4i), suggesting PTN may predominantly induces a transition to iCAF-related phenotype in HER2 + IBC.

In summary, by comparing the functional status and molecular heterogeneity of IBC and nIBC tumour mesenchymal stromal cells, we found significant differences in their subpopulation breakdown and functional status. There was also a significant increase in the proportions of inflammatory and antigen-presenting CAFs in the IBC tumour microenvironment, whereas the nIBC tumour microenvironment contained an abundance of myCAFs. Inflammation-related molecules and antigen presentation-related molecules were expressed at relatively high levels in IBC tumour-infiltrating mesenchymal cells, and in particular, the immune-responsive MHC class II antigen-presenting molecule CD74 was significantly overexpressed in specific subtypes. Therefore, these results indicate that the signals of immune cell activation, exogenous antigen presentation and angiogenesis are more active in IBC tumour-infiltrating mesenchymal cells, and the related molecules in these pathways may mediate the transition from the myCAF state to the inflammatory CAF state and promote the formation of the humoral immune microenvironment.

### HER2 + IBC tumour cells mediate extensive cellular interactions through high expression of the heparin-binding growth factor PTN

To compare the differences in interactions between cellular components within the IBC and nIBC tumour microenvironments, we assessed the interactions between seven main cell types. First, we compared the number of interactions between the various cell types and found that there was a general trend towards enhanced interactions in IBC tumours, with only epithelial and endothelial cells showing a decreasing trend in the number of interactions, whereas mesenchymal and myeloid cells had the most numerous and complex network of interactions (Fig. 5a). In terms of the overall number of interactions, significantly more interactions were inferred in IBC samples than in nIBC samples (Fig. 5a). We subsequently assessed the pattern of interactions on the basis of signalling pathways and found that IBC samples presented more added signalling pathways in the release signalling pattern, with PTN significantly increased in epithelial cells; MIF, ANNEXIN, ANGPT, PDGF and CSF significantly increased in mesenchymal cells; SPP1 and TNF significantly increased in myeloid cells; ANNEXIN, GRN, APRIL, ANNEXIN and IL16 significantly increased in T cells; and TNF and IL16 significantly increased in B cells (Fig. 5b). The receptive signalling patterns revealed that the PTN signals in the recipients were mainly epithelial and mesenchymal cells; the MIF signals were mainly B cells, T cells and myeloid cells; the SPP1 signals were mainly epithelial and mesenchymal cells; and the ANNEXIN signals were mainly myeloid cells (Fig. 5c). Our gene expression analysis of the relevant genes involved in the reciprocal signalling pathways that occur specifically in IBC revealed that the ligand PTN molecule was significantly highly expressed in IBC tumour cells, and its expression was also somewhat elevated in mesenchymal cells. Its receptor molecules (SDC1, SDC2, SDC3, SDC4, ITGAV, and NCL) were also expressed to a certain extent in various cell types (Extended Data Fig. S7a). The ligand SPP1 was expressed predominantly by myeloid cells, while its receptor molecules (CD44, ITGA4, ITGA5, ITGB1, and ITGB5) were also expressed to some extent in a variety of cell types (Extended Data Fig. S7a). The ligand IL16 was highly expressed specifically in IBC tumour B and T cells, whereas its receptor (CD4) was expressed mainly in myeloid cells (Extended Data Fig. S7a). The ligand TNFSF13B was highly expressed specifically in myeloid cells, whereas its receptor was expressed at high levels in B cells (TNFRSF13B) and antibody-secreting cells (TNFRSF17) (Extended Data Fig. S7a).

Further differences in receptor‒ligand interaction signalling were assessed in IBC and nIBC tumour microenvironment cell interactions. In the tumour cell-as-signal-releaser mode of interactions, we found that the receptor‒ligand pairs significantly increased in IBC tumour cell interactions were PTN‒NCL, MIF‒CD74 + CXCR4, MIF‒CD74 + CD44, and MDK‒NCL and that these receptor‒ligand pairs mediated a wide range of interactions between the tumour and other cell types (Fig. 5d). Among the patterns of interactions in which tumour cells act as signal receivers, we found that the receptor pairs that were significantly enhanced in IBC tumour cell interactions were PTN-SDC4, PTN-NCL, MDK-SDC4, MDK-NCL, and LGALS9-CD44, and these receptor pairs highlighted that endothelial, mesenchymal, and myeloid cells actively interact with tumour cells (Fig. 5d). By comparing the expression differences and correlations of PTN, MDK, and MIF, three ligand molecules that are more active in IBC tumour cells, we found that expression levels of PTN molecules were significantly upregulated in IBC tumour cells and that there was a significant positive correlation between the pathway scores of these three ligand molecules and the HER2 signature gene set scores (Extended Data Fig. S8a). Moreover, we performed a correlation analysis between the pathway scores of PTN, MDK, and MIF ligand molecules and the signature gene set scores of three important stages of epithelial cell development and found that the pathway scores of all three molecules were strongly correlated with tubular cell precursor status (Extended Data Fig. S8b). Immunohistochemistry experiments for PTN molecules were performed using paraffin samples of clinically sourced HER2 + IBC and nIBC tumour tissues, and we verified at the protein level that the expression of PTN molecules was greater in tumour tissues of IBC origin than in those of nIBC origin (Fig. 5e). In 26 IBC patients, high expression of PTN molecules was significantly positively correlated with better progression-free survival, and there was also a trend towards a positive correlation with overall survival (Fig. 5f). Since all patients with HER2 + IBC tumours received standard anti-HER2 + targeted therapy, there was a significant positive correlation between high expression of PTN molecules and high expression of Her2, indicating that high expression of PTN molecules could be used as a molecular marker for a better therapeutic response in patients with Her2 + IBC. We further correlated the expression levels of PTN receptor-related molecules with the signals of the functional status characteristics of the receptor cells and found that there was a strong positive correlation between the high expression levels of PTN receptor-related molecules and the high signals of the functional status characteristics of the receptor cells and a trend towards greater correlations in epithelial, mesenchymal, myeloid, and antibody-secreting cells of IBC (Extended Data Fig. S7b). We performed a similar correlation analysis of the expression levels of MIF receptor-related molecules and found a strong positive correlation between high expression levels of MIF receptor-related molecules and high signals characterizing the functional state of immune cells, with a trend towards greater correlations in B cells and antibody-secreting cells from IBC samples (Extended Data Fig. S7c). By comparing the expression of PTN in IBC tumour cells of different molecular subtypes, we found that PTN in HER2 + tumour cells had a moderate but significantly higher expression level, and there was a strong correlation between its expression level and the HER2 signature scores (Fig. 5g). The multiplex fluorescence results of tumour samples from HER2 + IBC patients also confirmed that PTN was mainly expressed in tumour cells (Extended Data Fig. S10a), while NCL had variable expression levels in various cell types, among which B cells and tumour cells were the main high-expression cell types of NCL (Extended Data Fig. S10b).

To explore the mechanism involved in making CD8 + T cells lose their cytotoxicity to tumour cells in HER2 + IBC microenvironment, we conducted a further analysis of the communication between CD8 + T cells and other types of cells. The results indicated that multiple cell types could interact with CD8 +/CD8 + Tex cells through the MIF-CD74 receptor-ligand pair, and the communication between tumour cells and CD8 + Tex cells was the strongest (Extended Data Fig. S11d). By conducting expression analysis on the main immune checkpoint molecules, we found that CD8 + T cells highly expressed LAG3 and TIGIT (Extended Data Fig. S11c). It has been reported that after MIF binds to the CD74/CD44 receptor, it can activate NFAT1 through the PI3K/AKT/MAPK signaling pathway, and NFAT1 can promote the expression of T-cell immune checkpoints including LAG3. Consistent with this result, the results of multiplex immunofluorescence experiments indicated that HER2 + tumour cells highly expressed MIF and presented a spatial neighborhood relationship with CD74 + CD8T cells (Fig. 5h, i). Pre-treatment with MAPK or Akt inhibitors followed by stimulation with recombinant MIF protein in PBMC-derived CD8 + T cells isolated from HER2 + IBC patients revealed that MIF induces NFAT1 nuclear translocation (from cytoplasm to nucleus) and upregulates LAG3 and TIGIT expression, a process partially mediated by p38 signaling (Fig. 5j, l, m; Extended Data Fig. S11e). Furthermore, primary tumour cells isolated from HER2 + IBC patient tissues were co-cultured with CD8 + T cells. We observed that HER2 + IBC cells promoted NFAT1 nuclear translocation (from cytoplasm to nucleus) and the elevated expression of LAG3 and TIGIT in CD8 + T cells, which was effectively blocked by a CD74-neutralizing antibody (Fig. 5k, Extended Data Fig. S11f). JASPAR motif analysis (Supplementary Table S3) and ChIP–qPCR experiments further confirmed that HER2 + IBC cells enhance the binding of NFAT1 to the promoters of LAG3 and TIGIT in CD8 + T cells (Fig. 5n). Furthermore, CD8 + T cells were co-cultured with HER2 + IBC tumour cells to assess cellular cytotoxicity. Notably, the addition of a CD74-neutralizing antibody significantly impaired the cytotoxic activity of CD8 + T cells in the co-culture system (Fig. 5o). These findings suggest that in HER2 + IBC, tumour-derived MIF interacts with CD74 on CD8⁺ T cells to activate p38 MAPK, which in turn drives NFAT1 nuclear translocation and transcriptional activation of LAG3 and TIGIT. This cascade ultimately leads to the loss of CD8⁺ T cell cytotoxicity in the immunosuppressive tumour microenvironment of HER2⁺ IBC.

On the basis of the differences in cellular interactions, we concluded that PTN-mediated interactions play an important role in the formation of an immunosuppressive microenvironment in IBC tumours (Extended Data Fig. S8c). These results suggest that the microenvironmental interactions triggered by tumour cells are more heterogeneous, whereas IBC tumour cells may promote the formation of an immunosuppressive microenvironment in HER2 + IBCs through PTN, and MIF may play an important regulatory role in the extrafollicular development of B cells.

### The inflammatory response is involved in the differentiation and invasion of HER2 + IBC tumour cells

**Fig. 6:**
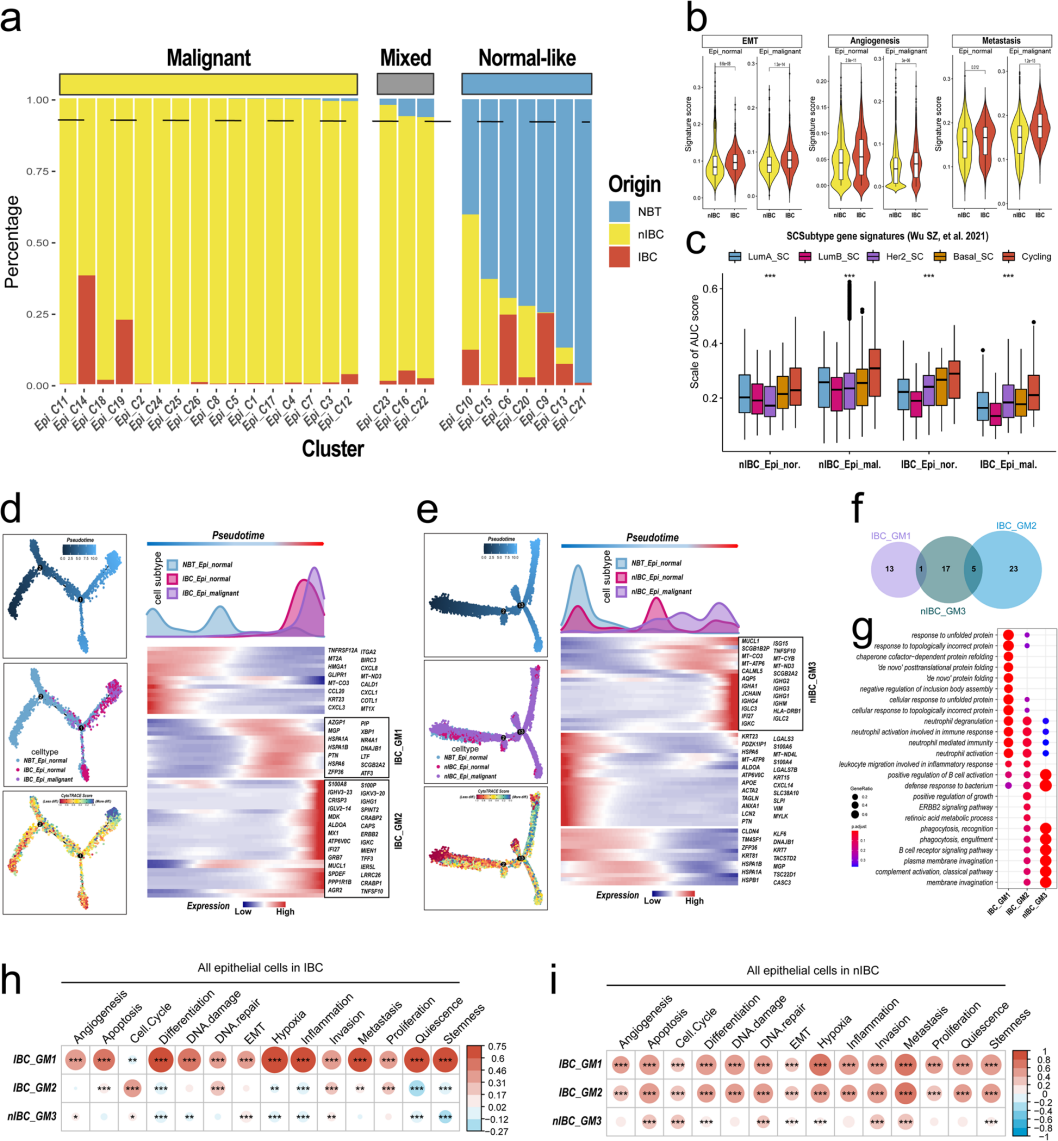
Molecular features of tumour cell differentiation. **a** Use of bar graphs to demonstrate the proportional distribution of different tissue origins in different epithelial cell subpopulations. **b** Use of violin plots to demonstrate the differences in 3 characteristic gene set scores (endothelial mesenchymal transition, invasion, and interferon response) between IBC and nIBC. **c** Demonstration of differences in 5 breast cancer single-cell signature gene set scores between IBC and nIBC tumour cells using box-and-line plots. **d-e** Proposed time-series analysis demonstrating differentiation trajectories of tumour cells in IBC (**d**) and nIBC (**e**). **f** Demonstration of gene sharing for the 3 tumour cell differentiation signature module gene sets using Venn diagrams. **g** Demonstration of GOBP enrichment for the 3 tumour cell differentiation signature module gene sets using bubble plots. **h-i** Demonstrate the correlation between the 3 tumour cell differentiation signature module gene set scores and the 14 tumour malignant molecular phenotype gene set scores using heatmaps in IBC (**h**) and nIBC (**i**), respectively

The name “inflammatory breast cancer” is defined from the description of clinical symptoms, and it remains controversial whether molecular pathways associated with the inflammatory response are involved in the malignant transformation of normal epithelial cells to cancer cells in inflammatory breast cancer. To compare the heterogeneity of epithelial cells and the differences in tumour cells from IBC and nIBC in more detail, we reclustered all epithelial cells into 26 subpopulations (Extended Data Fig. S9a). The distribution of epithelial cell subpopulations in tumour tissues and normal tissues was significantly different from the sample sources of different subpopulations (Fig. 6a). Therefore, we divided the different epithelial cell subpopulations into three major categories on the basis of the proportion of normal tissue sources in the epithelial cell subpopulations: normal-like epithelial cells (Epi_normal-like, NBT% > 10%: C6, C9, C10, C13, C15, C20, C21), mixed epithelial cells (Epi_mixed, 1% < NBT% < 10%: C16, C22, C23), and the remaining epithelial cell subpopulations were malignant epithelial cells (Epi_malignant, NBT% < 1%). From the UMAP plots of cell subpopulations, a more distinct spatial distribution of different epithelial cell subpopulations was observed (Extended Data Fig. S9b), and we evaluated the chromosomal variants of epithelial cells in IBC using CopyKat and inferCNV and found that malignant epithelial cells had a greater proportion of chromosomal aneuploidy variant conditions, indicating better concordance in the identification of tumour cells (Extended Data Fig. S9d). Subsequently, differential gene expression and pathway enrichment analyses were performed on all epithelial cells from different tissue sources, and the signalling pathways enriched for genes specifically expressed in IBC epithelial cells were found to be related mainly to cellular movement, including multicellular organismal movement, musculoskeletal movement, skeletal muscle contraction (extension), and skeletal muscle contraction (Extended Data Fig. S9c). The signalling pathways enriched in nIBC were associated mainly with chemokines, including those involved in cell chemotaxis, leukocyte chemotaxis, and the regulation of cell‒cell adhesion (Extended Data Fig. S9c). We further assessed the molecular phenotype of epithelial cells using the gene sets related to malignant tumour biology and found that the enrichment scores of three gene sets, EMT, invasion, and interferon alpha response, were significantly greater in IBC normal-like epithelial cells and malignant epithelial cells than in nIBC (Fig. 6b). Moreover, we performed a similar score comparison using the breast cancer single-cell molecular subtype signature gene sets and found that the cycling and Her2 signature gene set scores were higher than the other signature gene set scores in malignant epithelial cells (Fig. 6c).

To understand the process of malignant differentiation of tumour cells from normal epithelial cells, we performed pseudotime analysis of epithelial cells in IBC and nIBC samples. When epithelial cells derived from normal and IBC tissues were incorporated for pseudotime analysis, we found that epithelial cells derived from IBC tissues were predominantly in the tail of the pseudotime, and the pseudotime distribution of normal-like epithelial cells and malignant epithelial cells in the IBCs revealed an anterior and a posterior densitometric peak, which better fit the trajectory of malignant differentiation of the tumour cells (Fig. 6d). Using correlation analysis to identify expressed genes with pseudotime distribution correlation, we identified 2 gene modules (GM) with high kinetic correlation with the pseudotime distribution of epithelial cells in IBCs, which were named IBC_GM1 and IBC_GM2, in which IBC_GM1 was highly correlated with the pseudotime distribution status of normal-like epithelial cells, whereas IBC_GM2 was highly correlated with the pseudotime distribution state of malignant epithelial cells (Fig. 6d). On the basis of a similar approach, pseudotime analysis was performed on epithelial cells derived from normal and nIBC tissues, and we similarly found that the pseudotime results better fit the differentiation trajectory of epithelial cell malignancy in nIBC and that the pseudotime distribution of the normal-like epithelial cells in nIBC had anterior, middle, and posterior 3 density peaks, which demonstrated a more sequential process of malignant differentiation (Fig. 6e). In the identification of expressed genes associated with the pseudotime distribution, we found only 1 gene module (niBC_GM3) with a high kinetic correlation with the pseudotime distribution of epithelial cells in nIBC (Fig. 6e). Venn analysis of the 3 module gene sets revealed that nIBC_GM3 intersected most with IBC_GM2 with 5 genes (IGHG1, IGKC, IFI27, MUCL1, and TNFSF10) but shared only one gene, SCGB2A2, with IBC_GM1 (Fig. 6f). Similarly, GOBP enrichment analysis of the three modular gene sets revealed that nIBC_GM3 was more similar to the pathways enriched by IBC_GM2, whereas IBC was enriched for specifically activated pathways related mainly to the inflammatory response and cell proliferation, among which the inflammatory response-related pathways were neutrophil degranulation, neutrophil activation involved in the immune response, and neutrophil activation involved in the immune response (Fig. 6f). The pathways related to neutrophil activation involved in the immune response, neutrophil-mediated immunity, neutrophil activation, leukocyte migration involved in the inflammatory response, and cell proliferation-related pathways included positive regulation of growth, the ERBB2 signalling pathway, and the retinoic acid metabolic process (Fig. 6g). Notably, the inflammatory response signal had a strong activation state at the stage corresponding to IBC_GM1, suggesting that a strong inflammatory response accompanies the malignant transformation of IBC tumour cells at an early stage and persists until the malignant differentiation stage corresponding to IBC_GM2. We hypothesize that the inflammatory response may influence tumour cells undergoing different modes of malignant differentiation by altering their epigenetic modifications. When all epithelial cells were scored on the basis of a set of 20 histone modification-related genes, we found that the acetylation level of histone H3 was significantly greater in IBC tumour cells than in nIBC tumour cells (Extended Data Fig. S9e). The correlation between the 3-module gene set and the 14-tumour malignant molecular phenotype gene set was further assessed, and we found a significant positive correlation between the scoring of the IBC_GM1 module and most of the malignant molecular phenotypes of IBC tumour cells but a negative correlation between it and the cell cycle (Fig. 6h). Notably, the scores of the IBC_GM2 module were significantly positively correlated with the cell cycle, DNA repair, invasion and proliferation of IBC tumour cells, and significantly negatively correlated with differentiation and dormancy, suggesting that IBC tumour cells present different malignant molecular phenotypes at different stages of differentiation (Fig. 6h). In nIBC tumour cells, we found significant positive correlations between the 3-module gene set scores and most of the malignant molecular phenotypes, with two of the IBC-derived gene modules also showing significant positive correlations in nIBC tumour cells, suggesting that IBC and nIBC tumour cells share many similar molecular events during malignant differentiation (Fig. 6i). These results suggest that EMT signalling is highly activated in IBC tumour cells compared with HER2 + nIBC tumour cells, thus exhibiting a more pronounced malignant phenotype. Moreover, the highly invasive characteristics exhibited by IBC tumour cells may be correlated with malignant differentiation mediated by a sustained inflammatory response.

### TNF promotes the inflammatory response by mediating B-cell‒endothelial cell interactions in HER2 + IBC tumours

**Fig. 7:**
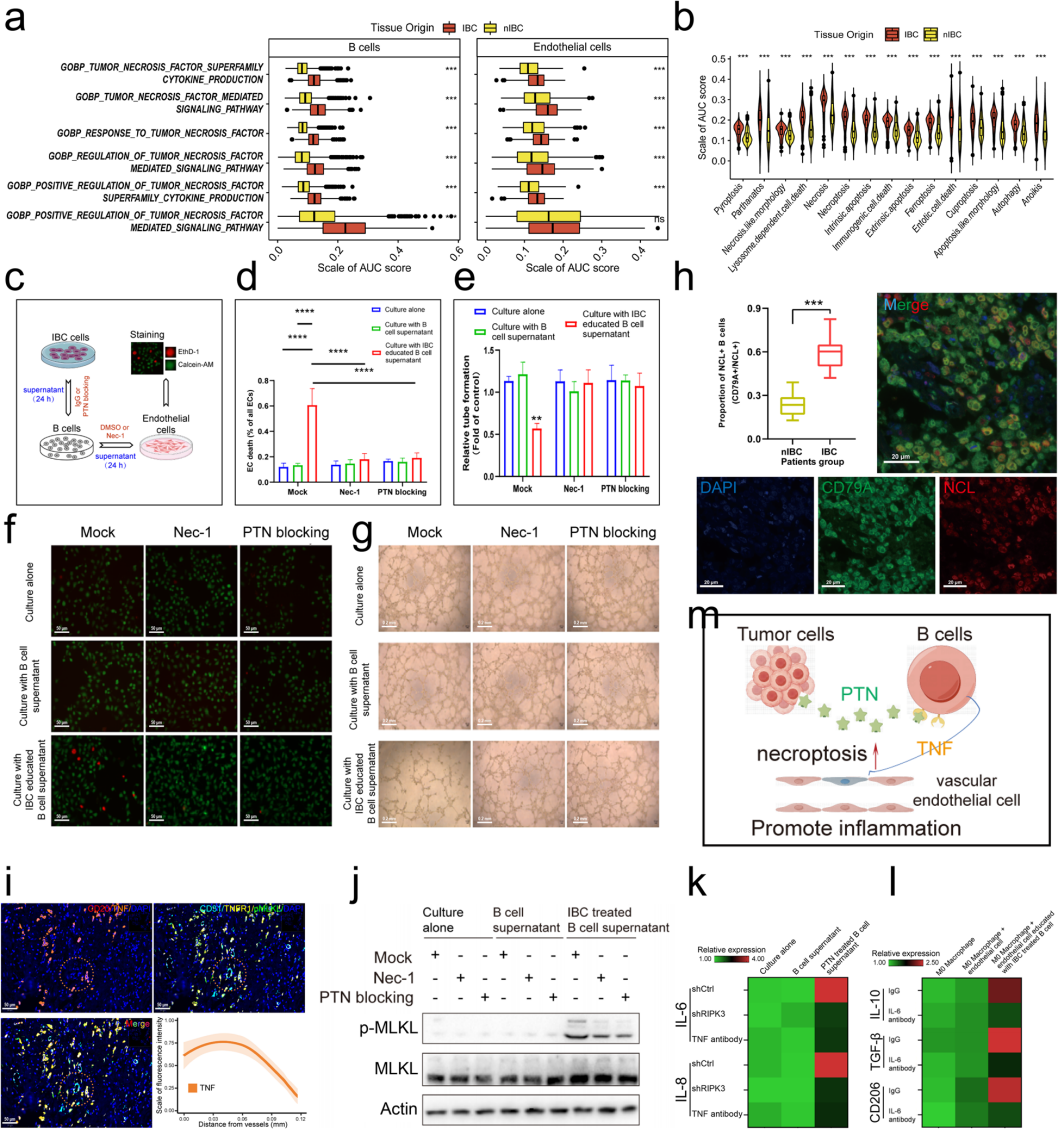
B cells in the IBC tumour microenvironment promote inflammatory responses through high expression of tumour necrosis factor. **a** Comparison of 6 TNF-related pathway signals in B cells and endothelial cells. **b** Comparison of 15 in death signalling scores in endothelial cells. **c** After co-culturing IBC-educated B cell supernatant with endothelial cells, the viability of endothelial cells was assessed by Calcein-AM/EthD-1 staining. The experimental schematic is shown. **d, f** Nec-1 or PTN blocking peptide was used to inhibit endothelial cell death induced by IBC-educated B cell supernatant. The bar graph (**d**) shows the percentage of endothelial cell death, and immunofluorescence imaging (**f**) shows the Calcein-AM + and EthD-1 + endothelial cells. **e, g** sIn endothelial tube formation assays, Nec-1 or PTN blocking peptide was used to block endothelial cell death induced by IBC-educated B cell supernatant. Quantification of tube formation is presented as bar graphs (**e**), and representative tube structures are shown by phase-contrast microscopy (**g**). **i** TNF + B cells were found around the necrotic endothelial cells based on multiplex immunofluorescence. **j** Under normal culture, B cell supernatant co-culture, or IBC tumor cell-treated B cell supernatant conditions, Nec-1 or PTN blocking peptide was used to block endothelial cell death induced by IBC-educated B cell supernatant. Western blotting was used to detect phosphorylated MLKL. **k** Under normal culture, B cell supernatant co-culture, or PTN-treated B cell supernatant co-culture conditions, RIPK3 expression in endothelial cells was knocked down or TNF neutralizing antibody was used to block endothelial cell death induced by IBC-educated B cell supernatant. q-PCR was used to measure the expression levels of IL-6 and IL-8 in endothelial cells. **l** M0 macrophages were cultured under normal conditions, endothelial cell supernatant co-culture, or supernatant of endothelial cells educated with IBC-treated B cells. IgG or IL-6 neutralizing antibody was added, and q-PCR was used to measure the expression of M2 macrophage markers (IL-10, TGF-β, and CD206) in M0 macrophages. **m** Diagram of the mechanism of interactions between tumour cells, B cells and endothelial cells in the IBC tumour microenvironment

The TNF-mediated inflammatory response is an important factor for the persistence of inflammatory signals in the tumour microenvironment. In a previous cellular interaction analysis, we found that Her2 + IBC tumour-infiltrating B cells presented high TNF signalling outputs and that their main receptor cells were endothelial cells (Fig. 5b, c). Therefore, we hypothesized that the inflammatory symptoms exhibited by IBC patients are related to TNF-mediated interactions between B cells and endothelial cells. First, when tumour-infiltrating B cells and endothelial cells were scored on the basis of a set of six TNF-related gene sets, we found that TNF-related pathway scores were higher in both IBC tumour-infiltrating B cells and endothelial cells than in nIBC, with the positive regulation of tumour necrosis factor mediated signalling pathway differences being most pronounced in B cells (Fig. 7a). Scoring tumour-infiltrating endothelial cells on the basis of 15 gene sets of cell death signals, we found that the various death signals were higher in IBC tumour-infiltrating endothelial cells than in nIBC, with the differences in necrotic and necroptotic signals being the most pronounced (Fig. 7b). Furthermore, the correlation between the receptor molecular signals of PTN and TNF signalling pathways was assessed in tumour-infiltrating B cells, and we found that the positive correlations between the receptor molecular signal scores of PTN and the scores of the three TNF-associated signalling pathways were greater in IBC tumour-infiltrating B cells than in nIBC, with the receptor molecular signal scores of PTN correlating with the response to tumour necrosis factor having the highest correlation between them (Extended Data Fig. S9f). Similarly, when we assessed the correlation between necroptotic signalling and TNF signalling pathways in tumour-infiltrating endothelial cells, we detected strong positive correlations between necroptotic signalling scores and all 3 TNF signalling pathway scores in both IBC and nIBC tumour-infiltrating endothelial cells (Extended Data Fig. S9g).

To confirm the above analysis results, we conducted the following experiments: The supernatant of B cells pretreated with PTN was co-cultured with endothelial cells, and then the viability and tube formation ability of endothelial cells were detected. The results indicated that B cells treated with PTN could significantly promote endothelial cells death (Extended Data Fig. S12b, d) and inhibit tube formation ability (Extended Data Fig. S12c, e). While interfering with the key kinase RIPK38 of the necroptosis pathway in endothelial cells or adding TNF neutralizing antibodies to the co-culture system could significantly inhibit this process (Extended Data Fig. S12c, e). Consistent with this result, supernatant of educated B cells by HER2 + IBC cells can significantly promote endothelial cell death (Fig. 7c, d, f) and inhibit tube formation ability (Extended Data Fig. S12a, Fig. 7e, g). However, treating endothelial cells with the RIPK1 inhibitor Nec-1 or adding PTN neutralizing antibodies during the co-culture of HER2 + IBC supernatant with B cells can significantly inhibit this process (Fig. 7d-g). Interestingly, inhibition of PTN-NCL-TNF signaling axis induced necroptosis of endothelial cells reduced HER2 + IBC cells migration over an endothelial cell layer (Extended Data Fig. S12f-j), which confirmed that HER2 + IBC tumour cells can mediate endothelial cell necroptosis through the TN-NCL-TNF signaling axis to promote tumour cell metastasis. In the tissue samples of tumour patients from clinical sources, we used multiplex immunofluorescence to confirm that the proportion of NCL + B cells in IBC was significantly higher than that in nIBC (Fig. 7h), and there was a clear co-localization between PTN + tumour cells and NCL + B cells (Extended Data Fig. S11b). At the same time, we observed that there were more TNF + B cells around necrotic blood vessels (Fig. 7i). These above results indicate that PTN released by HER2 + IBC cells can induce B cells to release TNF to mediate necroptosis of endothelial cells and inhibit tube formation ability, which promotes tumour metastasis. Under the stimulation of PTN, the results of the WB experiment confirmed that RIPK1-mediated MLKL phosphorylation was involved in the necroptosis of endothelial cells (Extended Data Fig. S12k). Under the stimulation condition of the supernatant of HER2 + IBC tumour cells, the results of the WB experiment can also confirm that RIPK1-mediated MLKL phosphorylation is involved in this process (Fig. 7j).

Necroptotic cells release a large number of inflammatory factors including IL-6, which can mediate the inflammatory response. IBC carcinoma tissues and serum are characterized by over-expression of IL-6 and the IBC animal model is characterized by high serum levels of IL-6 and IL-8 compared to non-inflammatory malignant mammary cancer. tumour associated macrophages (TAMs) are well-studied components of the TME in IBC, and preclinical studies in IBC have demonstrated the importance of TAMs, which was mediated by IL-6. Consistent with this, our experimental results indicate that HER2 + IBC tumour cells can mediate endothelial necroptosis through the PTN-NCL-TNF signaling axis to promote the expression of IL-6 and IL-8 (Fig. 7k). Subsequently, the endothelial cells were pretreated with IBC educated B cells for necroptosis induction. Then the supernatant of endothelial cells was co-cultured with M0 macrophages, and the expression of M2 macrophages markers (IL-10, TGF-β and CD206) was detected with qPCR. The results show that the expression of IL-10, TGF-β, and CD206 increased after treatment with supernatant of necroptotic endothelial cell, which was alleviated with IL-6 antibody blocking (Fig. 7l). In the clinically derived HER2 + IBC tumour tissues, we also observed that there were a large number of M2 macrophages around the necrotic endothelial cells, and at the same time, they had a high level of IL-6 expression (Extended Data Fig. S11a).These above results indicate that HER2 + IBC tumour cells may promote the differentiation of M2-type macrophages through endothelial necroptosis mediated by the TN-NCL-TNF signaling axis and lead to the rapid progression of HER2 + IBC.

**Fig. 8:**
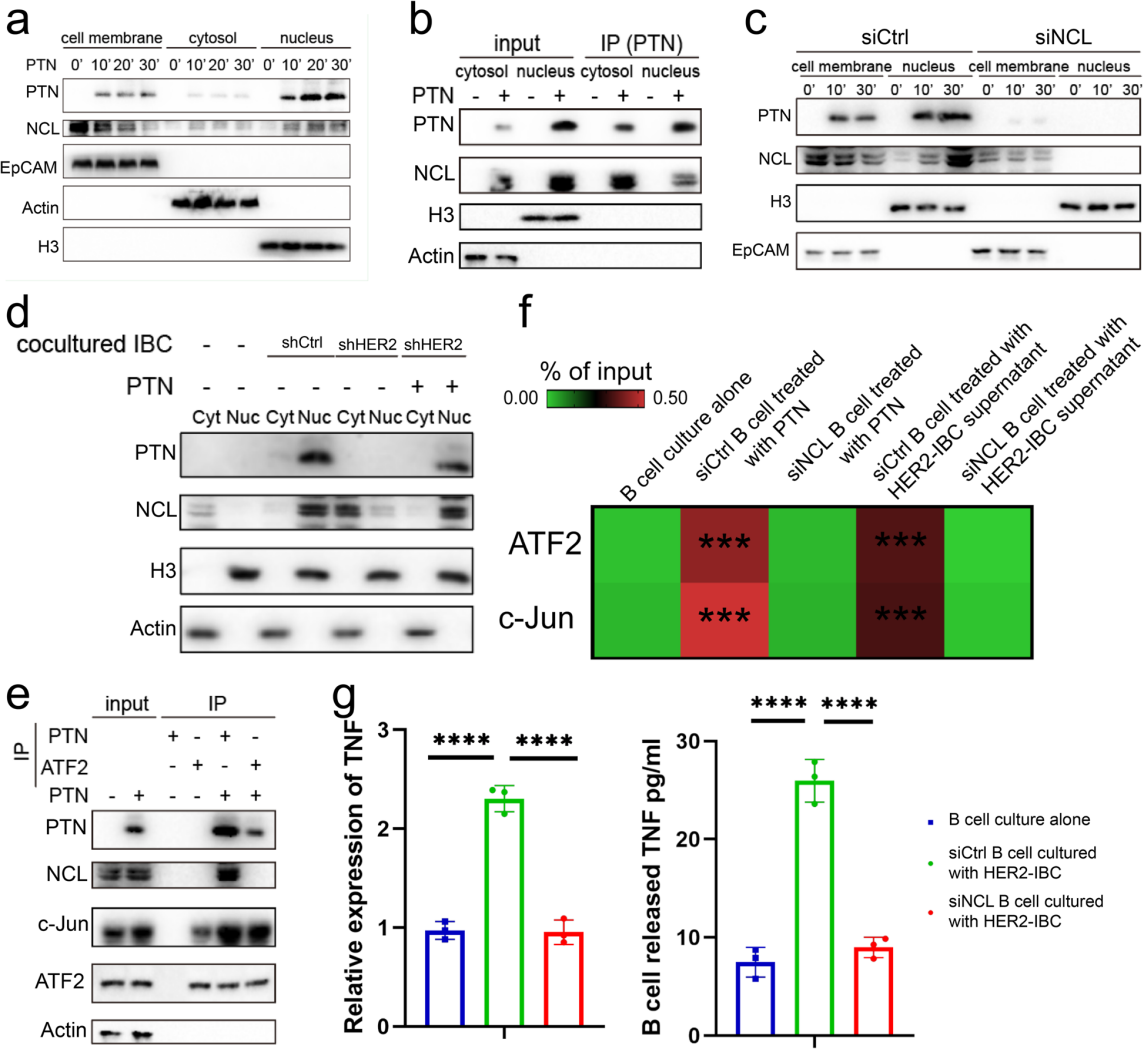
PTN Drives TNF Expression via NCL-Mediated ATF2/c-JUN Complex in B cells. **a** After treatment with PTN recombinant protein at different time points, Western blotting was used to detect the expression of PTN and NCL in the cell membrane, cytoplasm, and nucleus of B cells. **b** After treatment with PTN recombinant protein at different time points, immunoprecipitation and Western blotting were used to assess the binding of PTN and NCL in the cytoplasm and nucleus of B cells. **c** After interference with NCL expression, B cells were treated with PTN recombinant protein at different time points. Western blotting was used to detect the expression of PTN and NCL in the cell membrane and nucleus of B cells. **d** Control and HER2-interfered IBC tumor cell lines were co-cultured with B cells. DMSO or PTN was added to the co-culture system. Western blotting was used to detect the expression of PTN and NCL in the cell membrane and nucleus of B cells. **e** In control and PTN recombinant protein-treated B cells, immunoprecipitation and Western blotting were used to assess the binding of PTN, ATF2, and indicated proteins in B cells. **f** B cells were cultured normally or transfected with siCtrl or siNCL, and co-cultured with DMSO, PTN recombinant protein, or HER2-IBC supernatant. ChIP was used to detect the binding of ATF2 and c-Jun to the TNF promoter in B cells. **g** B cells were cultured normally or transfected with siCtrl or siNCL and co-cultured with HER2-IBC supernatant. q-PCR was used to measure the expression of TNF in B cells (left panel), and ELISA was used to detect the release of TNF in the B cell supernatant (right panel)

Finally, we explored the molecular mechanism by which HER2 + IBC cells promote the release of TNF by B cells. Previous studies have shown that HER2 can activate PI3K/AKT and MAPK signaling pathways either as a homodimer or as a heterodimer with other EGFR family members^1^⁸. These pathways lead to the activation of downstream transcription factors such as AP-1 and c-MYC. Specifically, the phosphorylation of Erk1/2 and p38 has been shown to mediate AP-1 activation, which in turn promotes the transcriptional activation of PTN^1^⁹. Consistent with the previous studies, we found that interfering with HER2 could inhibit the phosphorylation of p38 and Erk in HER2 + IBC tumour cells, as well as the expression of PTN (Extended Data Fig. S12l). After replenishing the continuously activated AP-1 subunit 5c-JUN^asp^, the expression of PTN after interference of HER2 could be patially rescued (Extended Data Fig. S12l). It has been reported that PTN can induce the expression of TNF in PBMC cells. To explore the molecular mechanism of TNF expression in B cells induced by PTN, we detected the subcellular localization of PTN and its receptor NCL at different time points after PTN treatment of B cells. We observed that upon PTN stimulation, PTN predominantly localized to the nucleus of B cells, while NCL translocated progressively from the plasma membrane through the cytoplasm into the nucleus (Fig. 8a). Co-immunoprecipitation assays demonstrated that PTN interacts with NCL primarily within the cytoplasm (Fig. 8b), suggesting that NCL may act as a shuttle, facilitating the nuclear translocation of PTN and subsequently releasing free NCL within the nucleus. Silencing NCL expression significantly impaired PTN nuclear translocation (Fig. 8c).

Further, western blot analysis confirmed that HER2 + IBC cells promote PTN nuclear import via NCL (Fig. 8d). Co-immunoprecipitation also revealed that PTN enhances the formation of an AP-1 transcriptional complex composed of c-Jun and ATF2 (Fig. 8e). JASPAR-based transcription factor binding prediction identified potential ATF2 and c-Jun binding sites within the TNF promoter region (Supplementary Table S3). Consistent with this, ChIP-qPCR assays showed increased binding of ATF2 and c-Jun to the TNF promoter following PTN stimulation of B cells or co-culture of B cells with HER2 + IBC cells; this effect was alleviated by NCL knockdown in B cells (Fig. 8f). qPCR and ELISA further confirmed that HER2 + IBC cells promote TNF expression and secretion of B cells via the PTN-NCL signaling axis (Fig. 8g). These results collectively suggest that the shuttling of NCL from the plasma membrane to the nucleus facilitates the nuclear delivery of PTN, which in turn enhances the formation of the c-Jun-ATF2 transcriptional complex, thereby promoting TNF transcription in B cells.

Taken together, these results suggest that tumour cells may promote the up-regulated expression of TNF in tumour-infiltrating B cells through release of PTN in the HER2 + IBC tumour microenvironment, then TNF can mediate necroptosis in tumour-infiltrating endothelial cells, which promotes both tumour cell metastasis and M2 macrophage polarization involved cancer progression (Fig. 7m). Moreover, elevated TNF signaling in tumour-infiltrating B cells may exacerbate the inflammatory response by promoting vascular damage, potentially contributing to the characteristic erythema elevatum observed in patients with IBC.

## Discussion

The unique molecular heterogeneity of the immunosuppressive microenvironment in inflammatory breast cancer [11], which poses a significant therapeutic challenge and partially explains the poor efficacy of immunotherapy in combination with chemotherapy or targeted therapies, is gradually gaining widespread attention among researchers [10]. Previous studies have attempted to elucidate major differences between IBC and nIBC by genomic and transcriptomic sequencing of tissue samples, but there is a lack of concordance in these findings [8, 39, 40], and it is still not possible to define molecular features specific to IBC in a better way. In this study, we constructed single-cell landscapes of the TME for the HER2 + molecular subtypes of IBC (3 patients, 15,832 cells) and nIBC (9 patients, 49,574 cells) while also integrating single-cell landscapes of normal breast tissue (5 patients, 14,767 cells) to identify the specific characteristics of the IBC TME. In terms of the cellular components of the tumour microenvironment, we found that the infiltration of lymphocytes in IBC tumours tended to be greater than that in normal tissues and nIBC tumours, with the most pronounced tendency to increase the proportion of infiltrating antibody-secreting cells. In terms of functional state shifts in major cell types, tumour cells in IBC presented increased interferon responses and EMT signals, T cells presented increased depletion and suppressive regulatory signals, B cells presented increased follicular epithelial responses, myeloid cells presented increased anti-inflammatory responses, and mesenchymal stromal cells presented increased antigen-presenting responses. Notably, we identified a subpopulation of antibody-secreting cells with SOX4 molecules as a characteristic marker, which also had a transcriptomic phenotype with high expression of inhibitory regulatory molecules. Importantly, we identified tumour cells of IBC that specifically express the heparin-binding growth factor PTN, the ligand for which is widely expressed in multiple cell types of IBC and the expression scores correlated strongly with the immunosuppressive status of the various cell types. These findings suggest that HER2 + IBC tumours have a greater ability to shape the immunosuppressive microenvironment, indicating that antibody-secreting cells with high expression of SOX4 molecules and tumour cells with high expression of PTN molecules need to be considered to develop more targeted therapeutic targets and adjuvant therapeutic regimens for the treatment of HER2 + IBC patients.

Our single-cell transcriptome profiling revealed a high percentage of lymphocyte infiltration in the microenvironment of HER2 + IBC tumours, which is more in line with the results of previous reports that a high percentage of lymphocyte infiltration is a more typical histopathological feature of IBC tumours [41, 42]. Among them, the proportion of infiltrating T lymphocytes was the highest, and the percentage of CD8 + T cells and effector memory T cells among all infiltrated T cells was also the highest, which indicated that the infiltrated T cells in HER2 + IBC tumours had a more active activation state. Moreover, the scores of genes related to the inhibitory regulation and exhaustion of T cells were also increased in IBC, and the molecules with increased expression levels included mainly FOXP3, CTLA4, and TIGIT, which also suggests that immunotherapy for IBC tumours needs to focus on other immune checkpoint molecules in addition to PD-L1 [43]. A high percentage of lymphocytic infiltration in IBC tumours is thought to be associated with a better response to neoadjuvant chemotherapy [44], but a stronger association has not been observed in cohort studies that have corrected for other prognostic factors [45]. A substantial decrease in the proportion of tumour-infiltrating lymphocytes has been reported in patients with IBC after neoadjuvant chemotherapy [46], which may be due to a more active immunogenic response induced by chemotherapy. Another report revealed that the absolute number of peripheral blood lymphocytes was significantly lower in patients with metastatic IBC than in healthy individuals [47]. On the basis of these findings, we hypothesize that the systemic immune system of IBC patients may have a certain disordered state; therefore, the treatment of IBC patients needs to focus not only on the infiltration characteristics of immune cells in the TME but also on the baseline level of the immune status of the patient’s peripheral blood to provide more validated biomarkers for accurate assessment of the benefit of immunotherapy. Notably, we found that the direction of activation and differentiation of infiltrating T cells in HER2 + IBC tumours was associated mainly with the NF-KB signalling pathway, which is characterized by high expression of heat shock protein-related molecules (HSPA1A, HSPA1B). One study defined T cells specifically overexpressing heat shock protein-associated molecules as being in a specific stress-responsive state, predominantly distributed in lymphocyte aggregates or potentially tertiary lymphoid structures at the tumour margins, and strongly associated with immunotherapy tolerance [17]. This further suggests that a better assessment of the benefit of immunotherapy in HER2 + IBC patients through the functional status of T cells is necessary.

We found that a high percentage of infiltrating B lymphocytes was also typical of the microenvironment of HER2 + IBC tumours, particularly a high percentage of antibody-secreting cells. A case report describing the initial clinical misdiagnosis of an IBC patient with plasma cell mastitis [48] suggested that the activation and differentiation of B cells in IBC tumours is more complex. The percentage of PD-L1-positive B cells has been reported to be an independent protective prognostic factor for overall survival and progression-free survival in IBC patients [44, 49], further emphasizing the need for a more comprehensive definition of the characteristics of infiltrating B cells in IBC patients. We found that B cells in HER2 + IBC tumours are more activated and highly express molecules characteristic of immature B cells, such as members of the Fc receptor family involved in immunoglobulin and immune complex binding. Among them, FCRL4 has been reported to be associated with tissue-resident memory cells and can inhibit BCR signalling [50]. FCGR2A represents a low-affinity inhibitory receptor that inhibits antibody-dependent cellular cytotoxicity (ADCC), antibody-dependent cellular phagocytosis (ADCP), and B-cell activation [51]. Moreover, we found that the important interferon molecule IFNG was highly expressed specifically in HER2 + IBC tumour-infiltrating B cells and antibody-secreting cells, suggesting a possible interfering role of IFNG in the normal activation and differentiation of B cells. It has been reported that IFNG mediates the activated differentiation of B cells undergoing alternative extrafollicular activation in some autoimmune diseases [52, 53]. Extrafollicular differentiation of B cells is a nonclassical differentiation pathway defined in recent years and exists predominantly in the form of a short-lived antibody-secreting cell state, which results in the production of antibodies that exhibit low-affinity and multiple reactivity characteristics [54, 55]. Moreover, a study investigating various tumour-infiltrating B cells revealed that enrichment of the extrafollicular differentiation state of B cells was associated with dysregulated immune responses and poorer clinical outcomes [56], suggesting that an atypical differentiation mode of B cells plays an important role in shaping the immunosuppressive microenvironment of tumours. Atypical memory B cells are the major progenitors of ASCs of extrafollicular differentiation origin, display depletion and bystander molecular phenotypes, and are independent of the developmental pathway of germinal centre differentiation, with representative expressed molecules notably including DUSP4, ITGAX (CD11c), FCRL5, ZEB2 and FGR [56]. We found a strong correlation between the expression of DUSP2 and DUSP5 in the direction of differentiation of HER2 + IBC tumour-infiltrating antibody-secreting cells and the putative chronology of differentiation by putative chronology analysis, which suggests that HER2 + IBC tumour-infiltrating B cells may contribute to the formation of an immunosuppressive microenvironment through a more active process of extrafollicular differentiation. We found that there was a group of specifically enriched cell subpopulations in HER2 + IBC tumour-infiltrating antibody-secreting cells, which were characterised mainly by high expression of SOX4, suggesting that the transcription factor SOX4 may play an important role in regulating the process of extrafollicular differentiation of B cells. Moreover, we found that high expression of SOX4 was associated with poor prognosis in most cancer types, suggesting that SOX4 may play an important role in multiple cell types of the TME. Current studies of SOX4 molecules in tumours have focused mainly on tumour cells and SOX4 molecules are thought to promote tumour progression by regulating the stemness characteristics of tumour cells [57, 58]. It has also been reported that SOX4 molecules can modulate the differentiation of Th-CXCL13 cell subpopulations, mediate the aggregation of B cells towards tertiary lymphoid structures, and promote better immune responses [59]. Therefore, we hypothesize that immunotherapeutic strategies targeting B cells may have a better therapeutic effect on HER2 + IBC tumours.

Through a detailed comparison of the heterogeneity of HER2 + IBC and nIBC tumour cells, we found that EMT, the interferon response and enrichment scores for invasive features were more strongly activated in IBC tumour cells, a finding that is more in line with previously reported results [60, 61]. Compared with their nIBC counterparts, IBC tumour cells have a greater invasive capacity, which is a consensus clinical feature, and all its molecular subtypes are associated with poorer response to treatment, recurrence-free survival and overall survival [6]. EMT is one of the important characteristic signals of tumours and plays an important role in invasion and early metastasis [62]; its increased activation in IBC is considered to be the most important signal for tumour invasion and early metastasis [62], and its increased activation in IBC is considered to be the most important in IBC. Higher activation is thought to be involved mainly in the formation of cancer plugs and dermal lymphatic vessels [63]. We found that two representative molecules involved in EMT, CD44 and HIF1A, were expressed at high levels in IBC tumour cells. CD44, a cell surface glycoprotein abundantly expressed in cancer stem cells [64], promotes cancer invasion and metastasis mainly by increasing cell motility [65–67]. HIF1A, a classical hypoxia-inducible factor subunit [68], induces the expression of matrix metalloproteinases [69] and promotes the degradation of the extracellular matrix, thereby increasing the invasion and metastasis of tumour cells [70]. The interferon response has a dual role in the tumour microenvironment, both promoting and suppressing tumours [71]. In general, the interferon response activates the immune system and promotes the trapping and killing of tumour cells by immune cells, but sustained low levels of the interferon response cause tumour cells to undergo adaptive transformation, which provides them with the opportunity to evade clearance by the immune system and achieve invasion and metastasis [72]. A study evaluating and comparing the degree of pathological complete response to preoperative chemotherapy in IBC patients revealed that IBC patients with high activation of the interferon response had a better degree of pathological complete response [73]; thus, therapeutic strategies targeting the interferon response in IBC patients may be beneficial. Through gene coexpression analysis, we revealed a coexpression relationship between the histone H3-3B gene and the CD44 and HIF1A molecules, and revealed that histone acetylation and deacetylation scores are increased in IBC tumour cells, which suggests that targeting the acetylation regulation of histones may be a potential therapeutic strategy. A study in which IBC tumour cells were screened for loss of gene function via RNA interference technology identified HDAC6 as an essential gene for maintaining IBC tumour cell viability, and an inhibitor of HDAC6 was found to be effective in controlling the value added to IBC tumour cells in a preclinical model [74]. Moreover, the combination of an HDAC inhibitor (entinostat) with HER2 + targeted therapeutic agents (lapatinib and trastuzumab) significantly enhanced the apoptosis of trastuzumab-resistant IBC tumour cells in a preclinical model [75]. Preliminary data from a subsequent phase Ib clinical study revealed that the addition of entinostat treatment to patients progressing on trastuzumab-based therapy improved the sensitivity to lapatinib and trastuzumab in both IBC patients and nIBC patients [7]. On the basis of the available research progress, we propose that in the malignant progression of HER2 + IBC tumour cells, the IBC-specific inflammatory environment promotes the active degree of histone acetylation in tumour cells, which in turn mediates the EMT transformation of IBC tumour cells and promotes invasion and metastasis; therefore, targeting the inflammation–histone acetylation–EMT signalling axis is necessary for IBC tumour therapeutic exploration.

We found that cellular interactions were significantly more active in the HER2 + IBC tumour microenvironment than in the nIBC tumour microenvironment, with particularly pronounced differences in cellular interactions mediated by heparin-binding growth factor (PTN) and macrophage-inhibitory factor for cell wandering (MIF). Heparin-binding growth factor (PTN)-secreting tubulointerstitial-like progenitor cells are more enriched in IBC patients and can mediate the proliferation of immature perivascular cells via the NRP1 receptor, thereby promoting the invasion and metastasis of IBC tumour cells [76]. Similarly, we found that PTN molecules were significantly overexpressed in HER2 + IBC tumour cells and strongly correlated with tumour EMT and invasive signals, suggesting that PTN molecules play important roles in IBC tumorigenesis and development. In other tumours, it has been reported that neural precursor cells can mediate glioma cell invasion towards the lateral to the subventricular zone (SVZ) by secreting PTN molecules [77]. Tumour-associated macrophages (TAMs) can also promote the malignant progression of glioma stem cells by secreting PTN molecules [78]. PTN molecules share high protein homology with MDK molecules, and together, they form a structurally unique family of heparin-binding growth factors [79] that play important roles in various physiological and pathological states [80]. In the present study, we also found that PTN molecule-mediated interactions play important regulatory roles in the functional polarisation of various stromal and immune cells in the HER2 + IBC tumour microenvironment and, in particular, correlate strongly with the polarisation of the inhibitory functional state of CAFs, TAMs and tumour-infiltrating ASCs. MIF is a multipurpose immunomodulatory cytokine, the main role of which is to inhibit randomly migrating immune cells, and it has been shown to be a good biomarker for the assessment of the inflammatory state in different diseases [81, 82]. In breast cancer, it has been reported that breast cancer stem cells can induce metabolic reprogramming of tumour cells by secreting MIF to generate an immunosuppressive microenvironment, suggesting that therapeutic strategies targeting MIF could improve the efficacy of breast cancer immunotherapy [83]. In this study, MIF-mediated cellular interactions were strongly correlated with the activated differentiation of B cells, especially the process of extrafollicular differentiation of B cells. Therefore, the present study revealed that PTN- and MIF-mediated signalling pathways play important roles in the formation of an immunosuppressive microenvironment in HER2 + IBC tumours, and therapeutic strategies targeting the corresponding pathways are expected to improve the efficacy of immunotherapy in HER2 + IBC patients.

These results highlight that patients with HER2 + IBC tumours have a more unique and complex tumour microenvironment. In particular, HER2 + IBC tumours have high levels of tumour necrosis factor (TNF)-related response signals in infiltrating B cells. On the basis of correlation analysis, we propose that HER2 + IBC tumour cells can influence the activation and differentiation process of B cells by upregulating the expression of the heparin-binding growth factor PTN, which in turn promotes the production of TNF by B cells. Moreover, TNF can act on endothelial cells and cause necrotic apoptosis of endothelial cells. These observations suggest that the blood vessels in the TME of HER2 + IBC tumours are at a greater risk of being abnormal, which is more in line with a recent study indicating that abnormal blood vessels are an important hallmark feature of tumours [84]. Compared with vessels in normal tissues, vessels in the TME often exhibit irregular morphology, poorer cellular arrangement, and increased vascular permeability [85]. On the basis of these findings, we propose that abnormal blood vessels can further promote inflammatory responses in the HER2 + IBC tumour microenvironment. Thus, in the TME of HER2 + IBC, abnormally active HER2 signalling can promote the release of the heparin-binding growth factor PTN from tumour cells, whereas PTN can act on B cells to promote the release of TNF from B cells, which in turn promotes necrotic apoptosis of endothelial cells to cause vascular abnormalities and ultimately promotes the inflammatory response. Moreover, the inflammatory response in turn promotes the malignant progression of tumour cells, and malignant tumour cells upregulate the expression of PTN. In this way, the PTN-TNF molecular axis-mediated cellular interactions create a vicious cycle in the HER2 + IBC tumour microenvironment centred on the inflammatory response, which prevents the formation of a more immunosuppressive microenvironment.

Our single-cell transcriptomic analysis revealed that in the TME of HER2⁺ IBC, the dominant population of activated immune cells comprised tumour-infiltrating T cells exhibiting a dual molecular phenotype characterized by high activation and high exhaustion. Specifically, the proportions of both CD8⁺ naïve T cells and CD8⁺ exhausted T cells were significantly elevated in IBC tumours compared to non-IBC tumours (Fig. 2c). Moreover, CD8⁺ naïve T cells in IBC exhibited enhanced activation signatures, including responses to interferon-γ, T cell activation, and neutrophil-mediated immunity, alongside an upregulation of protein folding-related pathways such as chaperone-mediated folding and de novo protein folding (Fig. 2h), suggesting that increased protein synthesis supports heightened T cell immune responses. In parallel, CD8⁺ exhausted T cells in IBC displayed enriched gene programs associated with mitochondrial energy metabolism, including ATP biosynthesis, purine ribonucleoside triphosphate production, and mitochondrial cristae formation (Fig. 2i).

Despite this apparent activation, we identified tumour-specific immunoregulatory interactions that may impair cytotoxic T cell function in IBC. Compared to non-IBC, HER2⁺ IBC tumour cells engaged CD8⁺ T cells through the MIF–CD74 ligand–receptor axis, while CD8⁺ T cells specifically upregulated immune checkpoint molecules LAG3 and TIGIT. Multiplex immunofluorescence further confirmed that HER2⁺ tumour cells highly expressed MIF and were spatially proximal to CD74⁺ CD8⁺ T cells (Fig. 5h, i). Functional assays demonstrated that neutralization of CD74 significantly impaired the cytotoxic activity of CD8⁺ T cells in co-culture (Fig. 5o). Mechanistically, HER2⁺ IBC cells promoted NFAT1 binding to the promoters of LAG3 and TIGIT in CD8⁺ T cells (Fig. 5j–n). These findings collectively suggest that in HER2⁺ IBC, tumour-derived MIF activates CD74 on CD8⁺ T cells, triggering p38 MAPK signalling and NFAT1 nuclear translocation, which drives transcriptional activation of LAG3 and TIGIT. This cascade leads to functional exhaustion and cytotoxic impairment of CD8⁺ T cells in the immunosuppressive microenvironment of HER2⁺ IBC. Thus, analyzing this “immune activation-inhibition” contradiction provide targets for HER2 + IBC immunotherapy (such as LAG3 and TIGIT), and our discovery may reshape the microenvironment through combined therapy to improve therapeutic effect and overcome drug resistance of HER2 + IBC.

## Conclusions

Overall, our study revealed the characteristics and molecular phenotypes of the cellular components of the immunosuppressive TME in patients with HER2 + IBC tumours and identified a signalling pathway for the formation of the immunosuppressive TME. Since IBC patients are relatively rare, this study was limited due to the low number of HER2 + IBC patients. However, our study provides a database and theoretical guidelines for the study of the TME of HER2 + IBC patients, which may lead to the discovery of more effective therapies targeting the formation of the immunosuppressive TME.

## Supplementary Information

Supplementary Material 1.

Supplementary Material 2.

Supplementary Material 3.

Supplementary Material 4.

## Data Availability

Data generated in this study are available within the article and its supplementary data files and are available upon request from the corresponding author. The datasets generated in this study are publicly available in GEO database (GSE161529, GSE164898, GSE176078, GSE161529).

## Abbreviations

IBC: Inflammatory breast cancer
nIBC: Non-inflammatory breast cancer
TME: Tumour microenvironment
scRNA: Single-cell transcriptome technology
ASC: Antibody secreting cells
HER2: Human epidermal growth factor receptor 2
PTN: Pleiotrophin
EMT: Epithelial mesenchymal transition
TNF: Tumour necrosis factor
GEO: Gene expression omnibus
PBS: Phosphate buffered saline
PCA: Principal component analysis
CNV: Copy number variation
GO: Gene ontology
GOBP: Gene ontology biological process
KEGG: Encyclopedia of genes and genomes
GSEA: Gene set enrichment analysis
FFPE: Formalin-fixed paraffin-embedded
TBS: Tris-Buffered Saline
DAPI: 4’,6’-Diamidino-2-phenylindole
DAB: Diaminobenzidine
CD4_Tcm: CD4 + central memory T-cells
CD4_Treg: CD4 + regulatory T cells
CD8_TNaive: CD8 + naive T cells
CD8_Trm: CD8 + tissue-resident memory T cells
CD8_Tem: CD8 + effector memory T cells
CD8_Tex: CD8 + exhausted T cells
Proli_T: Proliferating T cells
NKT: Natural killer T cells
MHC: Major histocompatibility complex
VDJ: The variable, diverse and Joining gene segments of the antibody
BCR: B cell receptor
CAF: Cancer-associated fibroblasts
BRCA: Breast invasive carcinoma
TCGA: The cancer genome atlas
Macro: Macrophages
Mono: Monocytes
cDC: Classical dendritic cells
iDC: Inflammatory dendritic cells
iCAF: Inflammatory CAF
myCAF: Myofibroblast-like CAF
apCAF: Antigen-presenting CAF
NF: Normal-like fibroblasts
GM: Gene module
MIF: Macrophage-inhibitory factor for cell wandering
SVZ: Subventricular zone
TAM: Tumour-associated macrophages
