# Supplementary material for: Single-cell transcriptome analysis reveals the malignant characteristics of tumour cells and the immunosuppressive landscape in HER2-positive inflammatory breast cancer
Source: J Exp Clin Cancer Res. 2025 Jul 8;44:196. doi: 10.1186/s13046-025-03454-z (PMC12235857; doi:10.1186/s13046-025-03454-z)
Supplement: Supplementary file 1 — Supplementary Material 1. [file 13046_2025_3454_MOESM1_ESM.docx]

**Title:**

**Single-cell transcriptome analysis reveals malignant characteristics of tumor cells and immunosuppressive landscape in HER2 positive inflammatory breast cancer**

**Supplemental figures and legends**


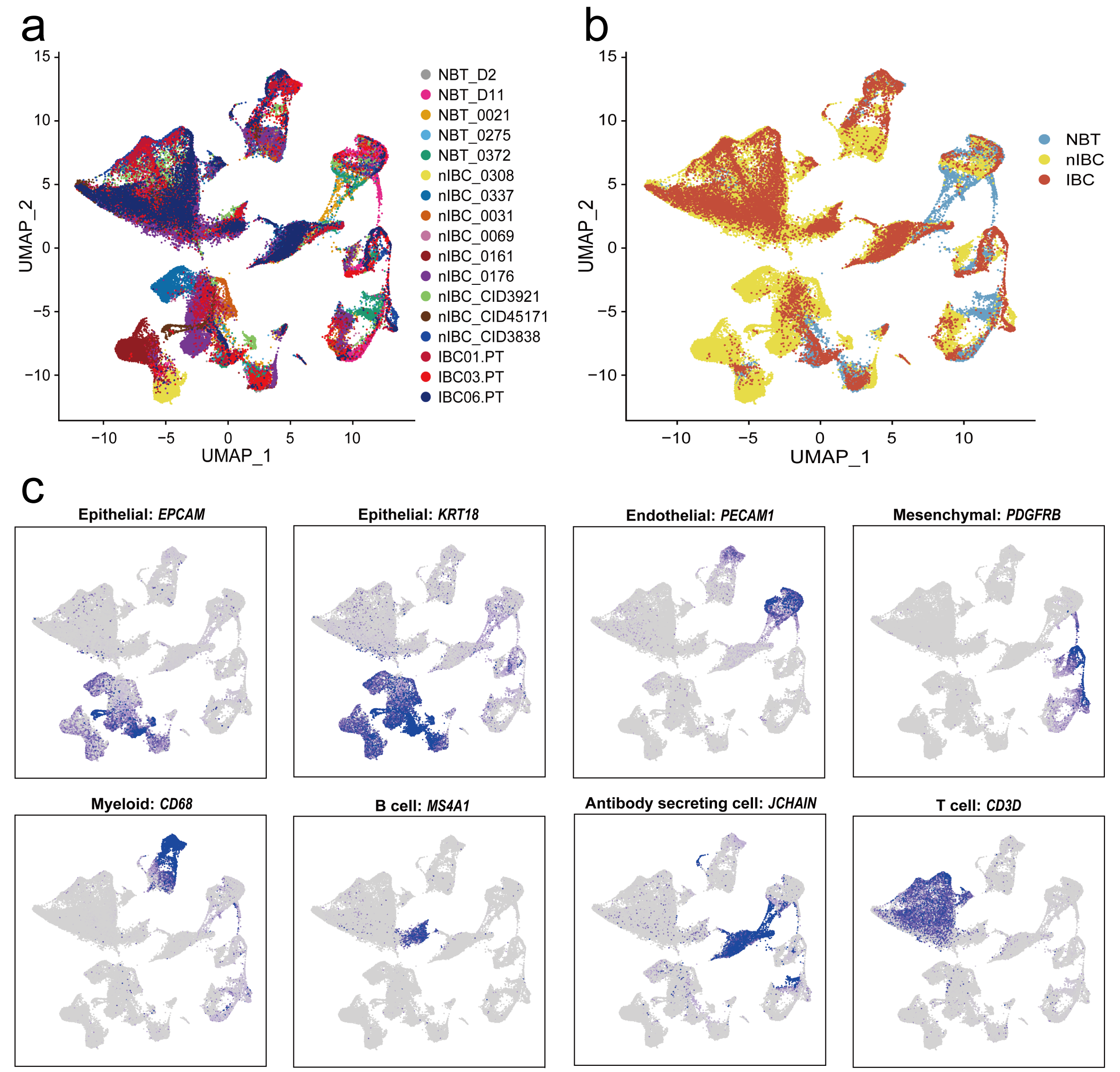


**Figure S1. Supplementary data for the single-cell transcriptome landscape for inflammatory breast cancer.**

**a-b** Demonstration of the integration of single-cell datasets using UMAP plots at the patient (**a**) and sample tissue source (**b**) levels, respectively.

**c** Demonstrates the expression of marker genes in the major cell types using UMAP plots.


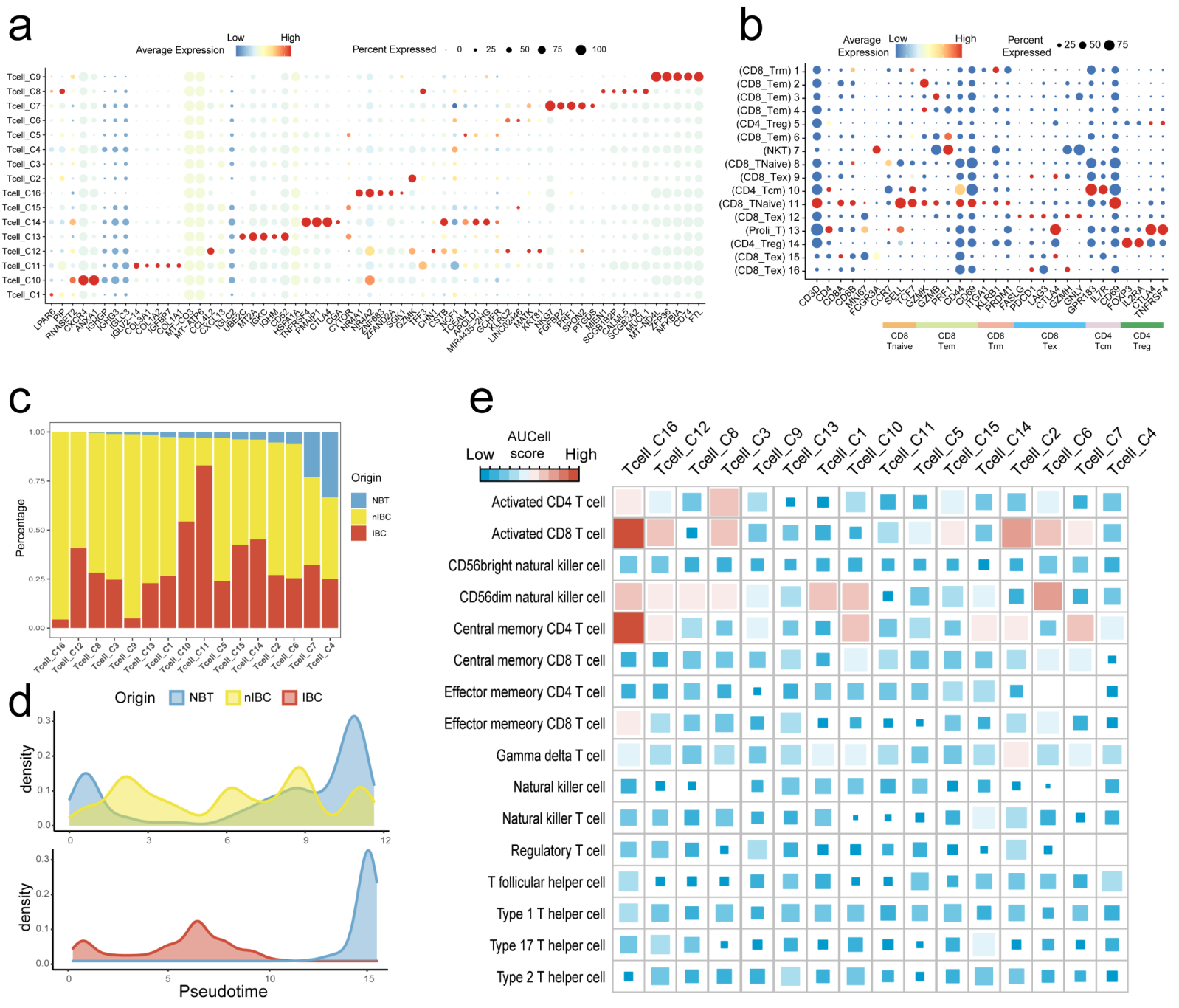


**Figure S2. Supplementary data on T-cell heterogeneity.**

**a** Demonstration of relevant genes specifically highly expressed in different T cell subpopulations using bubble plots.

**b** Demonstration of the expression of CD4+ T-cell-related genes and CD8+ T-cell-related genes in different T-cell subpopulations using bubble plots, respectively.

**c** Demonstrates the proportional distribution of tissue types in different T cell subpopulations using bar charts.

**d** Demonstrate the dynamic plasticity of T cells in IBCs and nIBCs using densitograms.

**e** Using heatmaps to demonstrate signalling differences in T-cell-associated gene set scores in different T-cell subpopulations.


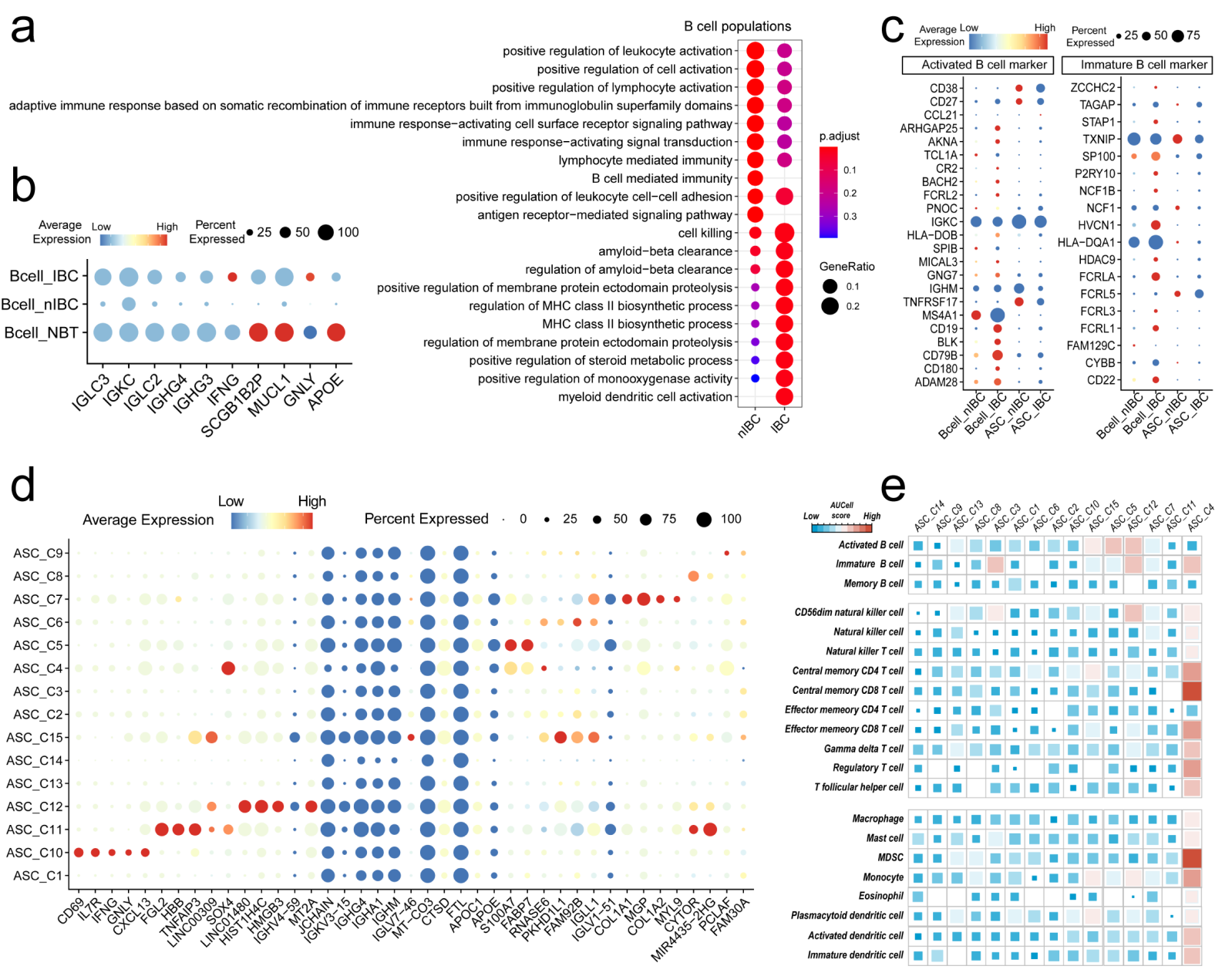


**Figure S3. Supplementary data on the heterogeneity of B cells and antibody-secreting cells.**

**a** Use of bubble plots to demonstrate GOBP enrichment of differentially expressed genes between IBC and nIBC by plasmoblasts.

**b** Demonstration of B cell and plasmoblast-specific highly expressed genes in IBCs using bubble plots.

**c** Using bubble plots to demonstrate the expression of B-cell activation-associated genes and B-cell immaturity-associated genes in B cells and plasmoblasts.

**d** Demonstration of relevant genes specifically highly expressed in different plasmoblast subpopulations using bubble graphs.

**e** Use of heatmaps to demonstrate signalling differences in immune cell-associated gene set scores in different plasmoblast subpopulations.


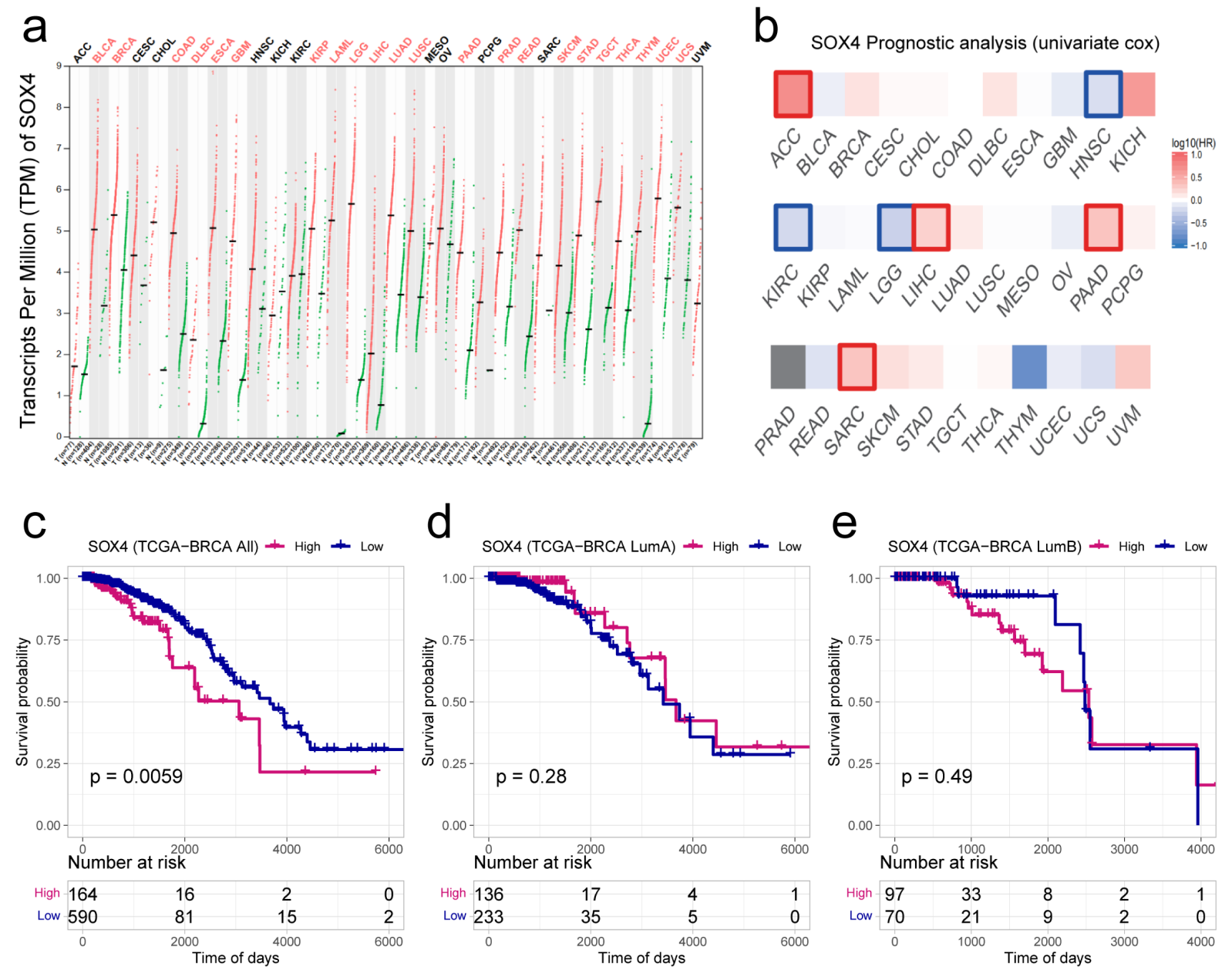


**Figure S4. Supplementary data on the prognostic significance of the SOX4 gene.**

**a** SOX4 gene expression in pan-cancer.

**b** Prognostic significance of SOX4 gene in pan-cancer.

**c-e** The relationship between the expression of the SOX4 gene and prognosis in different molecular subtypes was demonstrated by survival curves.


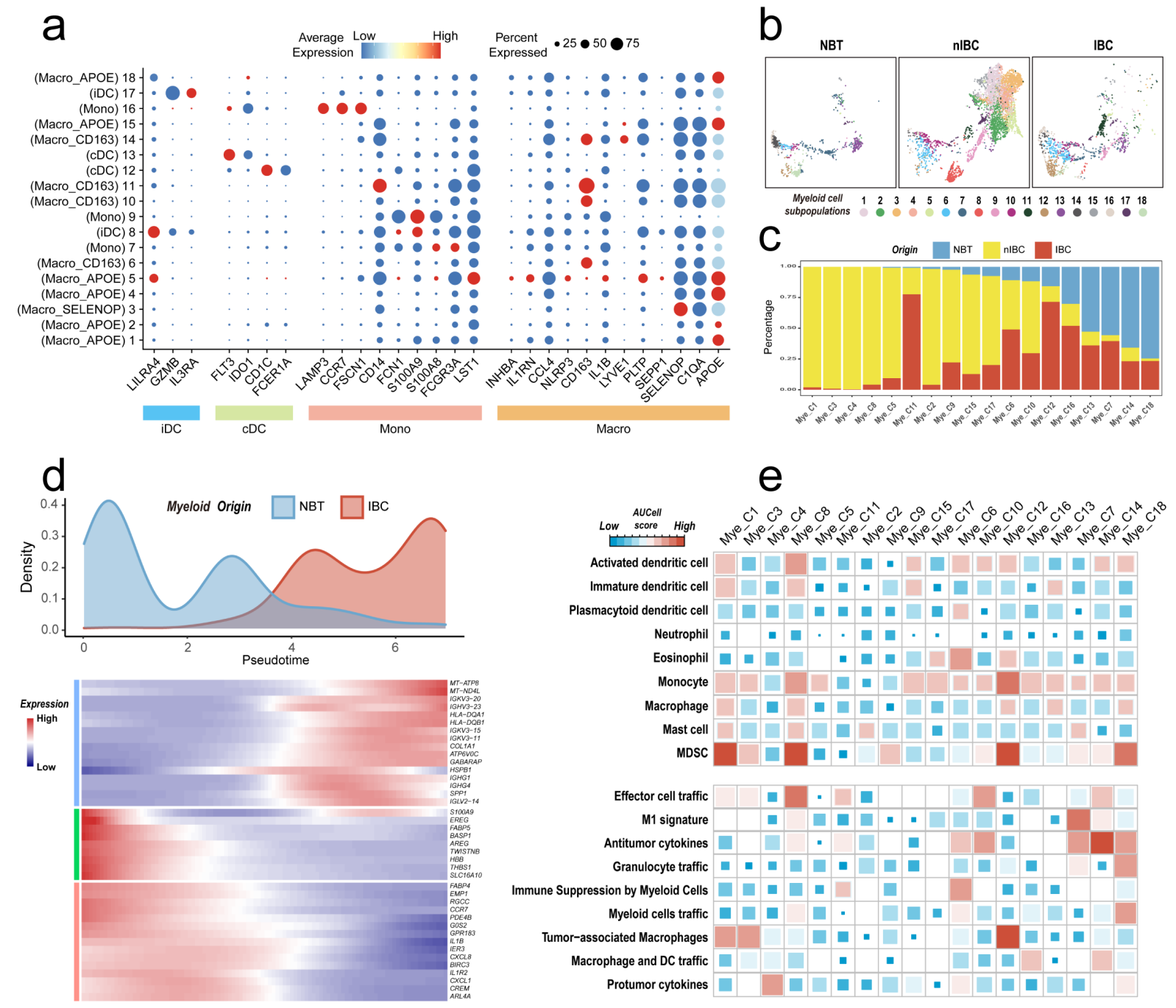


**Figure S5. Supplementary data on myeloid cell heterogeneity.**

**a** The expression of molecular markers related to myeloid cells in each cell subpopulation was displayed using bubble plots.

**b** Demonstration of the distribution of unsupervised clustered groups of myeloid cells using UMAP plots.

**c** Proportionate distribution of tissue types in different myeloid cell subpopulations demonstrated using bar charts.

**d** Proposed time series analysis of myeloid cells in IBC.

**e** Demonstration of signalling differences in myeloid cell-associated gene set scores in different myeloid cell subpopulations using heatmaps.


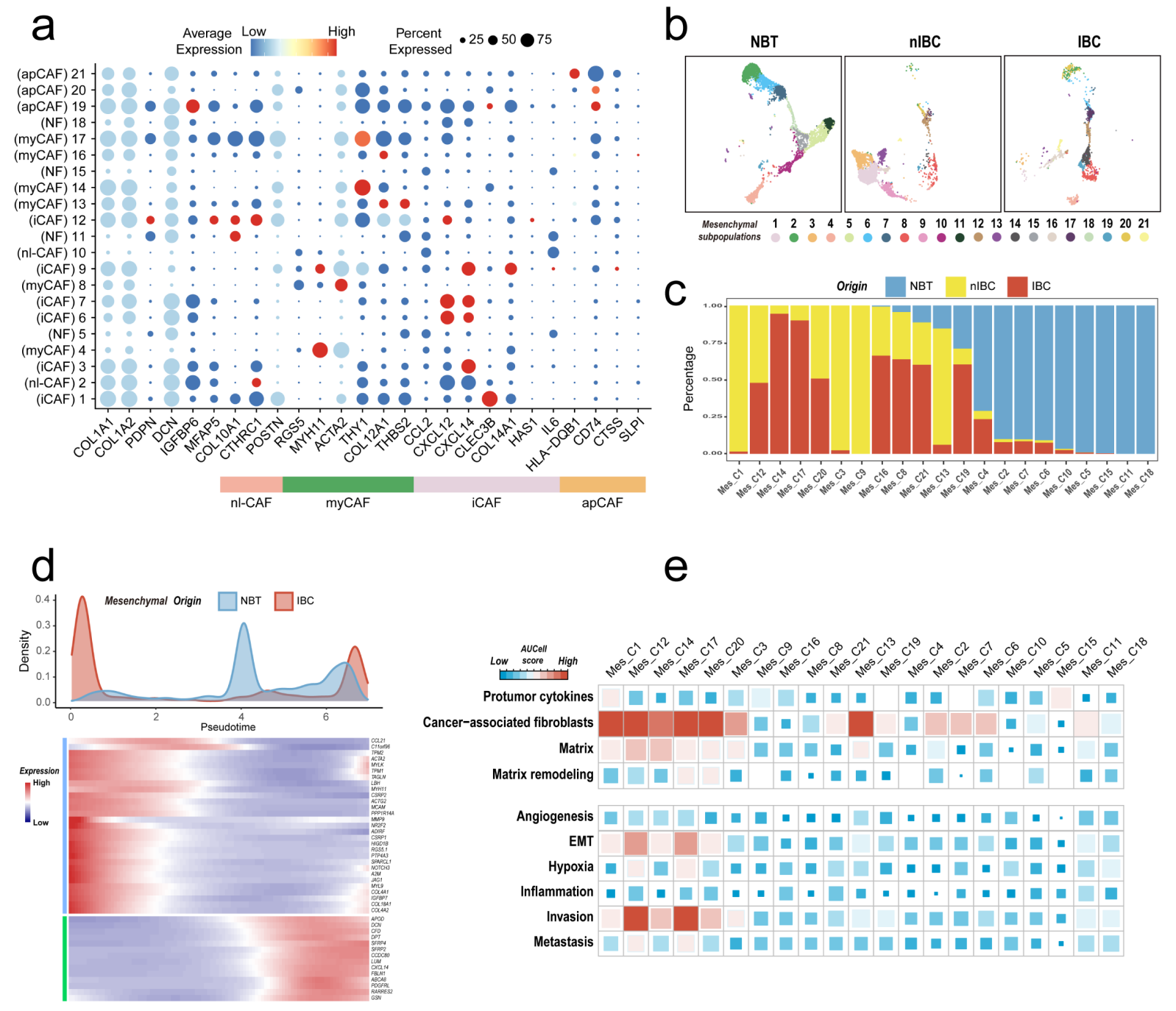


**Figure S6. Supplementary data on mesenchymal cell heterogeneity.**

**a** The expression of molecular markers related to mesenchymal cells in each cell subpopulation was displayed using bubble plots.

**b** Demonstration of the distribution of unsupervised clustering groups of mesenchymal cells using UMAP plots.

**c** Demonstrating the proportional distribution of tissue types in different mesenchymal cell subpopulations using bar charts.

**d** Proposed time series analysis of IBC mesenchymal stromal cells.

**e** Demonstration of signalling differences in tumour microenvironment-associated gene set scores in different mesenchymal cell subpopulations using heatmaps.


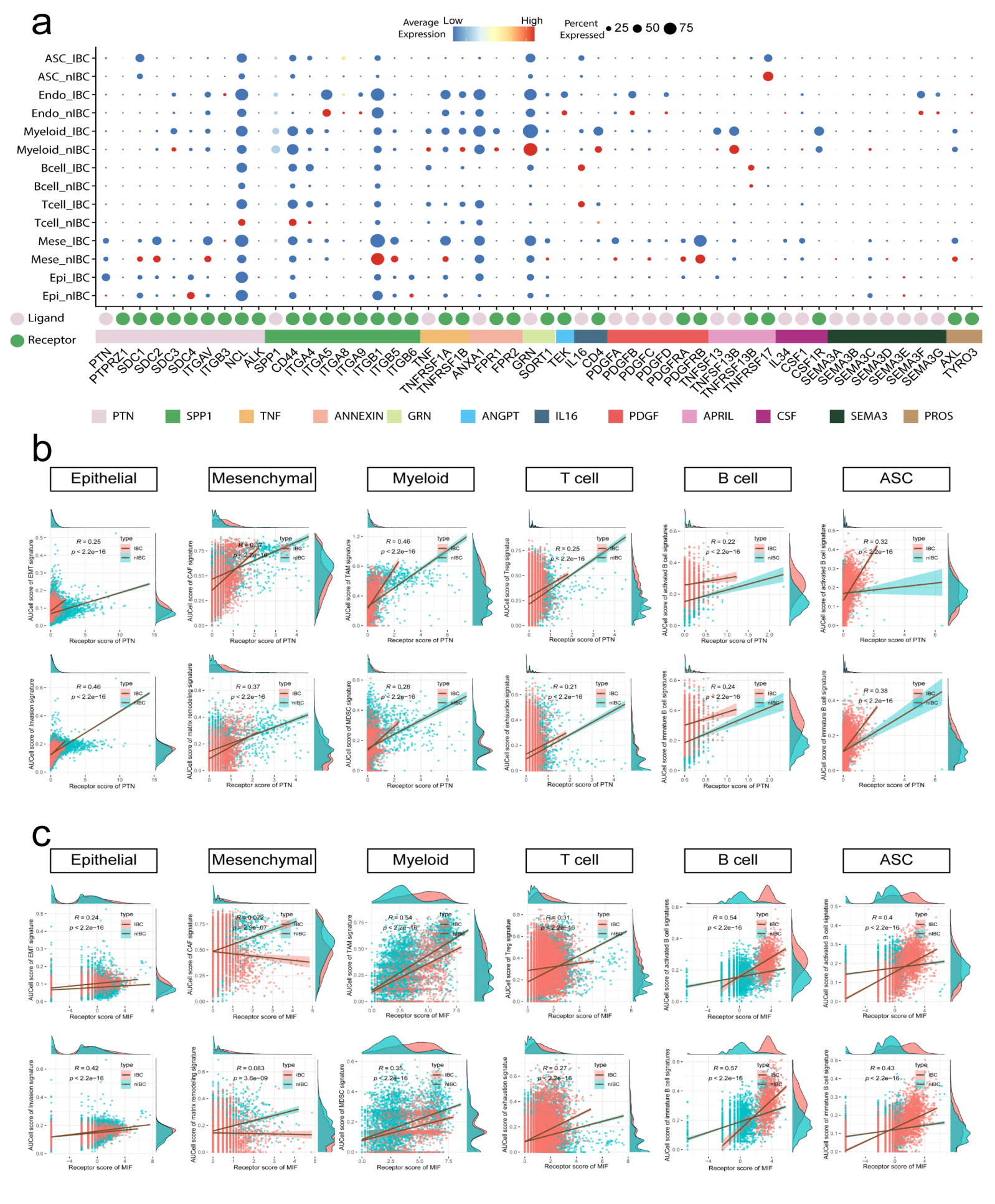


**Figure S7. Supplementary data for comparison of cellular communication.**

**a** Bubble plots were utilised to demonstrate the differences in expression of significantly different receptor ligand molecules in the major cell types of IBC and nIBC.

**b** Correlation between PTN receptor scores and major cell type functional polarisation feature scores is demonstrated using scatter plots.

**c** Correlation between PTN receptor scores and major cell type functional polarisation feature scores is demonstrated using scatter plots.


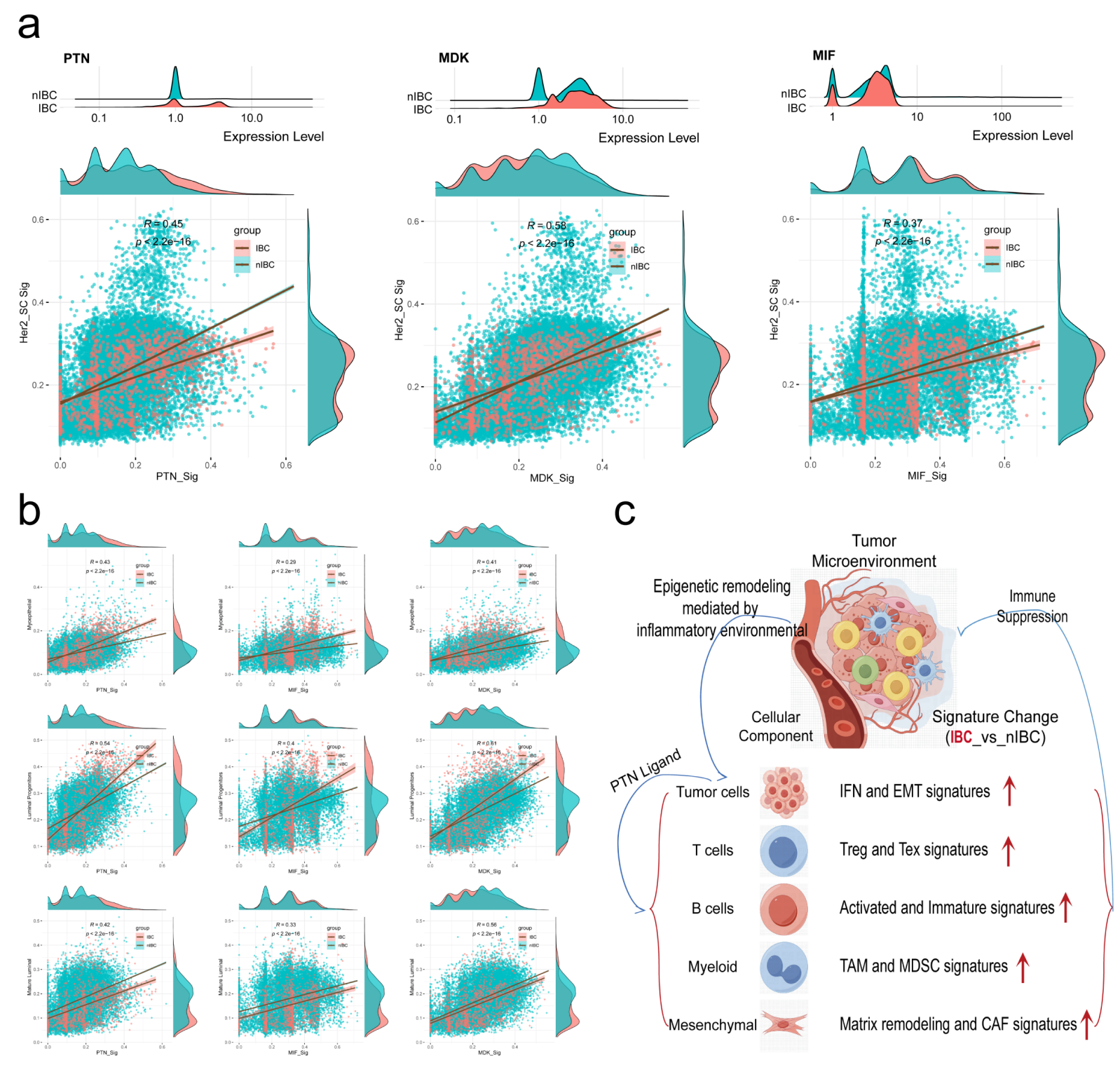


**Figure S8. Supplementary data for comparison of cellular communication.**

**a** Differences in the expression of the three molecules PTN, MDK, MIF between IBCs and nIBCs and the correlation with the Her2 signature gene set were demonstrated using ridge plots and scatter plots.

**b** Correlation between the feature scores of the three ligand molecules (PTN, MDK, MIF) and the scores of the three epithelial cell development and differentiation feature gene sets is shown using scatter plots, respectively.

**c** Pattern diagram of the ligand molecule PTN in IBC mediating the formation of an immunosuppressive microenvironment.


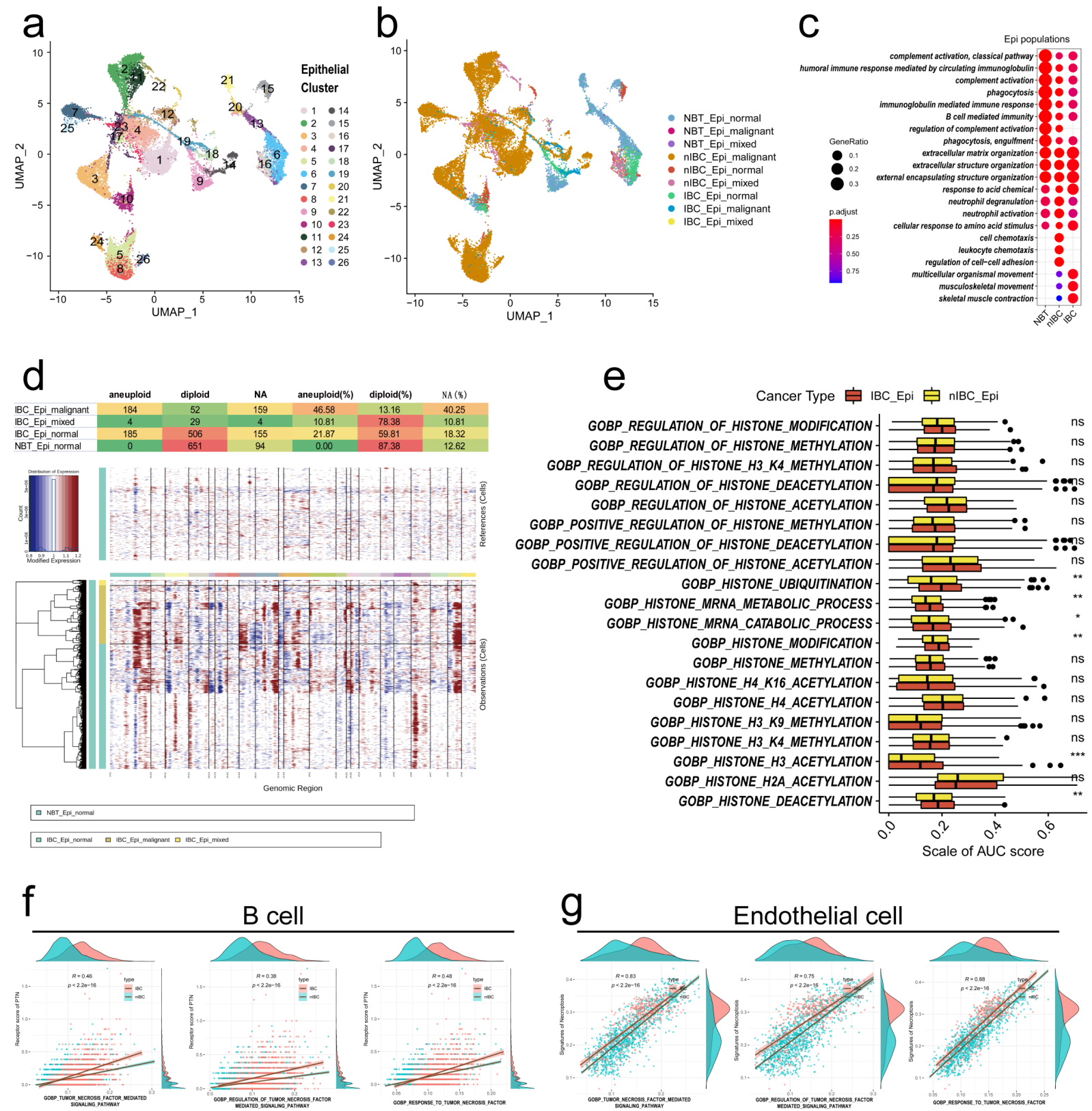


**Figure S9. Supplementary data on molecular characterisation of tumour cells.**

**a-b** Demonstration of the distribution of unsupervised clusters of epithelial cells (**a**) and clusters of malignancy (**b**) using UMAP plots.

**c** GOBP enrichment of differentially expressed genes among NBT, IBC and nIBC in epithelial cells is demonstrated using bubble plots.

**d** The assessment of chromosomal copy number variation in IBC tumour cells by CopyKat and inferCNV was demonstrated using heatmaps, respectively.

**e** Differences in gene set scores associated with 20 histone modifications between IBC and nIBC were compared using box-and-line plots.

**f** Correlation between PTN receptor scores and 3 TNF-related pathway scores in B cells is demonstrated using a scatter plot.

**g** Correlation between necroptosis apoptosis score and 3 TNF-related pathway scores in endothelial cells is demonstrated using scatter plots.


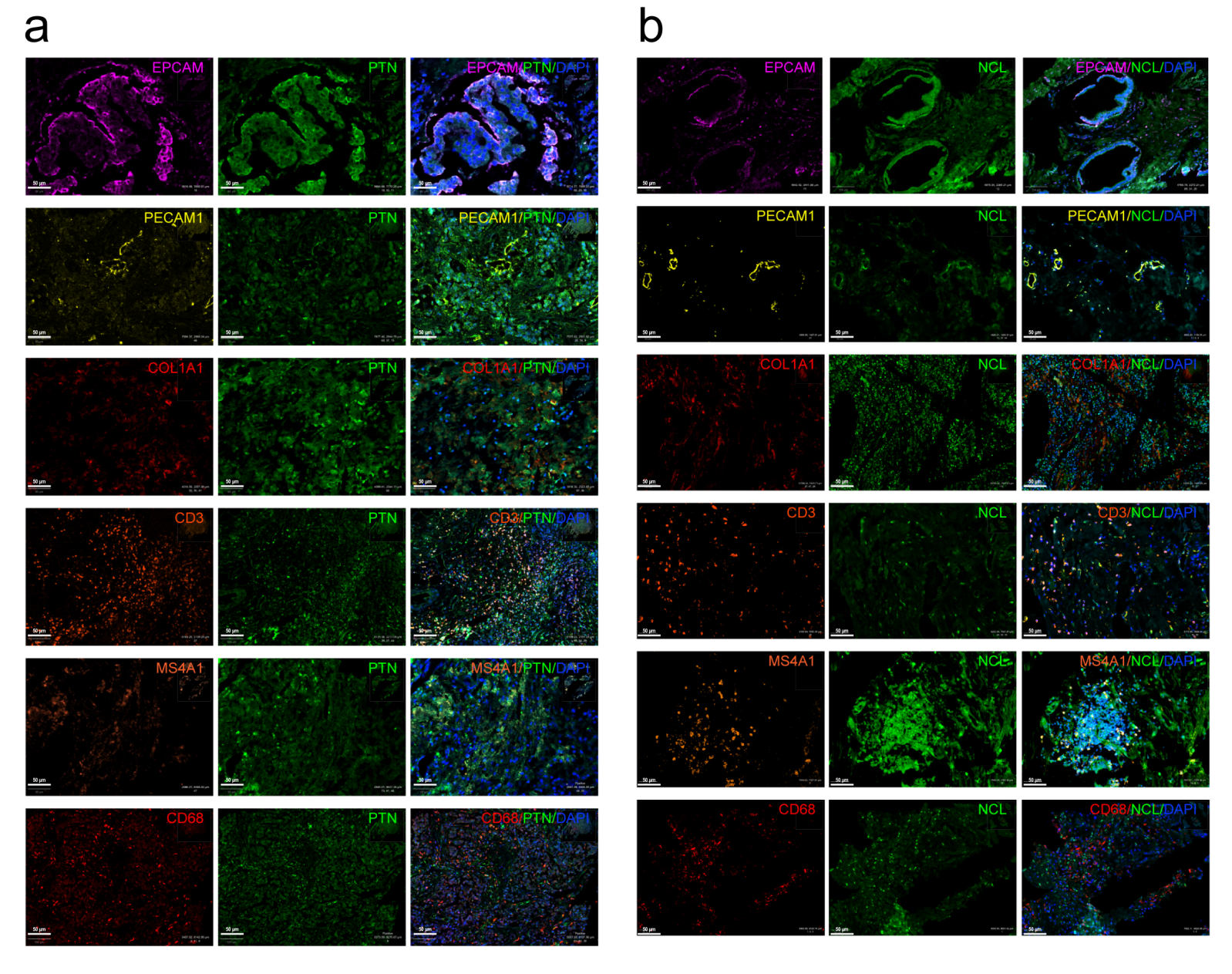


**Figure S10. Supplementary data on molecular characterisation of cellular interaction.**

**a** The expression of PTN molecules in various cell types was verified by multicolor immunofluorescence.

**b** The expression of NCL molecules in various cell types was verified by multicolor immunofluorescence.


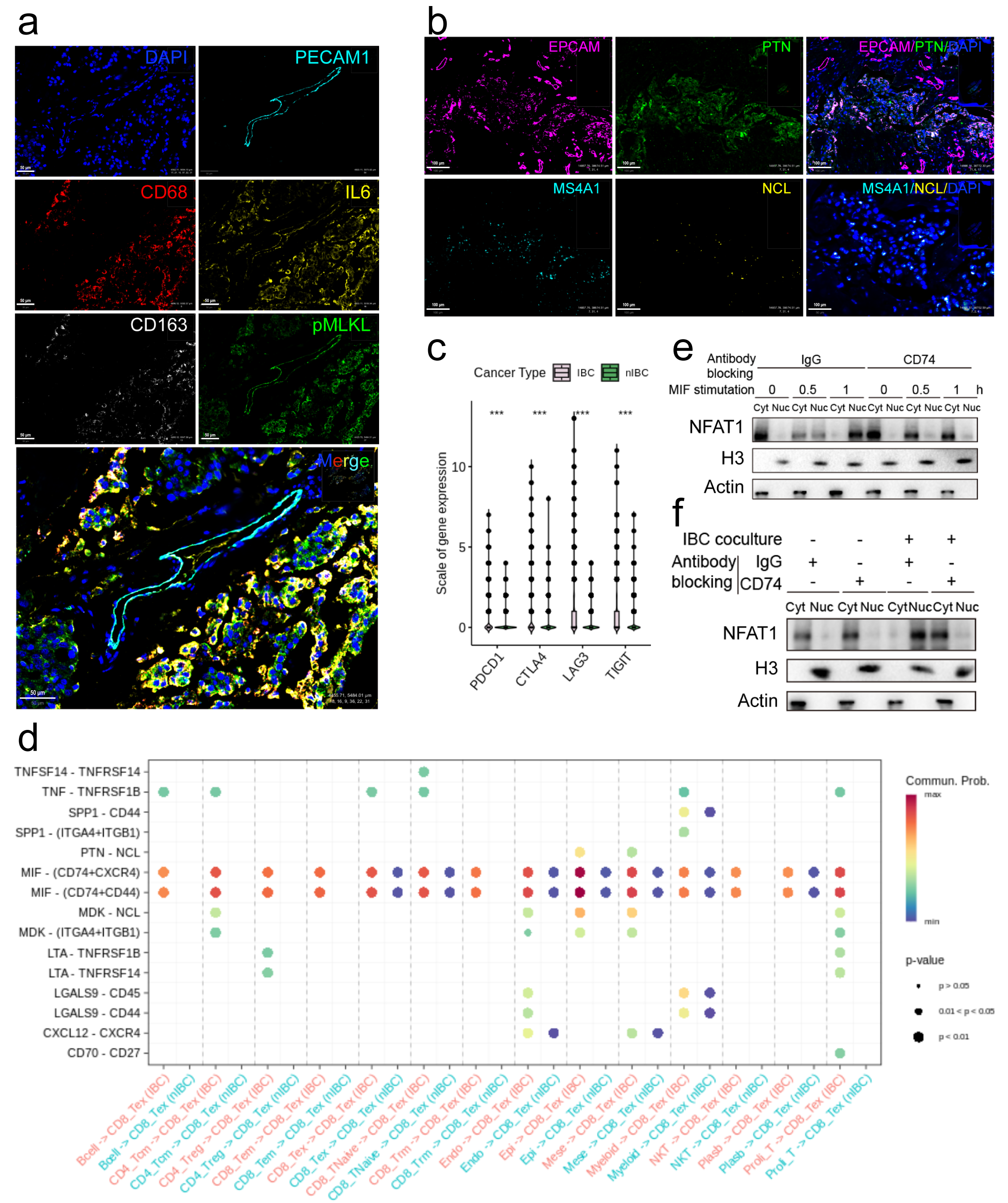


**Figure S11. Supplementary data on molecular characterisation of cellular interaction.**

**a** M2-type macrophages were found around the necrotic blood vessels based on multicolor immunofluorescence.

**b** There is a co-localization phenomenon between PTN+ tumor cells and NCL+ endothelial cells.

**c** The expression differences of four immune checkpoint molecules in IBC and nIBC tumor cells.

**d** Demonstrate the strength of ligand gene pairs with significant differences in cellular interactions using bubble plots.

**e** CD8+ T cells were stimulated with recombinant MIF for various time points in the presence of IgG or CD74 neutralizing antibody pre-incubated in the culture medium. Western blotting was performed to assess the cytoplasmic and nuclear distribution of NFAT1.

**f** CD8+ T cells were co-cultured with IBC tumor cells for indicated durations in the presence of IgG or CD74 neutralizing antibody. Western blotting was used to analyze the subcellular distribution of NFAT1 in T cells.


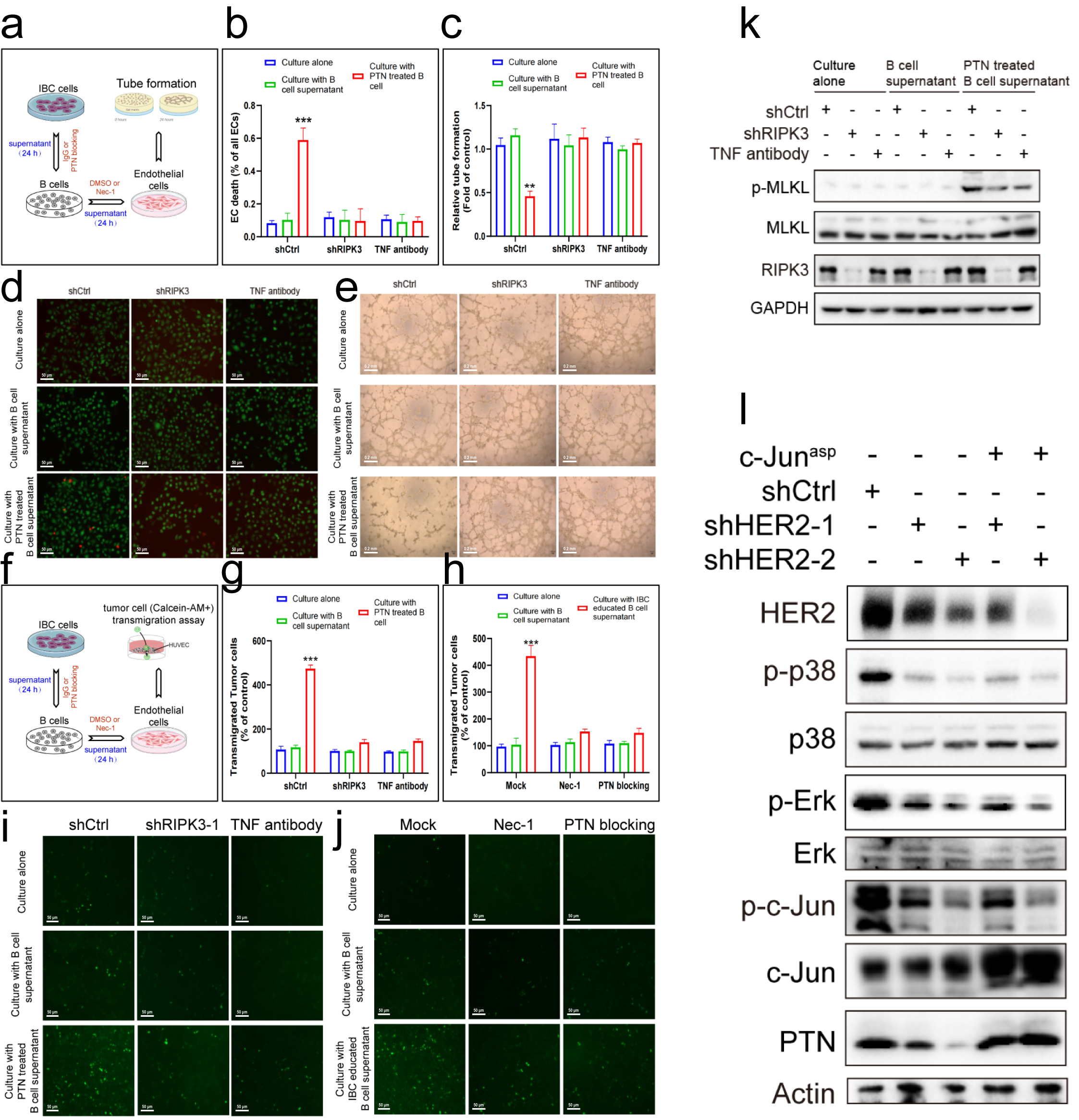


**Figure S12. Supplementary data on molecular characterisation of cellular interaction.**

**a** Schematic illustration of endothelial tube formation assays following co-culture with IBC-educated B cell supernatant.

**b, d** Under conditions of normal culture, co-culture with B cell supernatant, or co-culture with PTN-treated B cell supernatant, endothelial cells were subjected to RIPK3 knockdown or TNF neutralizing antibody treatment to block cell death induced by IBC-educated B cell supernatant. Bar graphs (**b**) show the proportion of endothelial cell death, and immunofluorescence (**d**) images illustrate Calcein-AM+ and EthD-1+ cells.

**c, e** Endothelial tube formation assays were performed under the same culture conditions as in S12b, d. RIPK3 knockdown or TNF blockade was applied to mitigate endothelial cell death induced by PTN-treated B cell supernatant. Quantification of tube formation is shown in bar graphs (**c**), and representative tube structures are displayed by phase-contrast microscopy (**e**).

**f** Schematic diagram of a transwell migration assay in which IBC tumor cells were seeded in the upper chamber directly above the endothelial cell monolayer that had been pretreated with IBC-educated B cell supernatant, to assess tumor cell transmigration across the endothelial barrier.

**g-j** Transmigration assays were performed under various culture conditions. Bar graphs (**g, h**) depict the percentage of migrated IBC tumor cells, and immunofluorescence images (**i, j**) show the transmigrated tumor cells.

**k** Western blotting of phosphorylated MLKL in endothelial cells cultured under normal conditions, co-culture with B cell supernatant, or co-culture with PTN-treated B cell supernatant, following RIPK3 knockdown or TNF neutralization.

**l** HER2 expression was silenced in HER2+ IBC cells, and constitutively active c-Junasp was reintroduced to evaluate the reactivation of MAPK signaling and c-Jun phosphorylation with western blotting.
