# Supplementary material for: Single-cell transcriptome analysis reveals the malignant characteristics of tumour cells and the immunosuppressive landscape in HER2-positive inflammatory breast cancer
Source: J Exp Clin Cancer Res. 2025 Jul 8;44:196. doi: 10.1186/s13046-025-03454-z (PMC12235857; doi:10.1186/s13046-025-03454-z)
Supplement: Supplementary file 4 — Supplementary Material 4. [file 13046_2025_3454_MOESM4_ESM.docx]

LAG3 promoter (NFAT1 binding sequences were yellow highlighted, while ChIP primer specific sequences were red font):

-1530
CAAAAGGGTAGAGAGGGAGGAGGGTGGCTGACCTCCCATTCTGCCAGGACCCTTCCCTTTGATACAAAAGCAGCAGCACACTCCTACCACCCACGACTCAAGAAGACAAGATCAGCCCTCCAGGGGTGGCCTGGAGCCCAGCTCTTCTGAGAGCTGACTTATGTCCTGTTTTGGATTCTCAGGGATGCTCCTCCCCAGCAATACACACTTCCCTGCTAACCCACACAAGACCTAGACACTGCTGAGCCCAGTGGAAAGCTACCAGCTCTATTGTGCTGAACCAGGCACAGAGAAACAGCCCAGAAACAGGAAGTCCTTCCCCTGAGCTGGGAGAGGGCTGGTAGTCCTGGGTCTCAGCCTGCTAGGATCTGGAGTGAGCTGCCTGGCATCAGAGATGCCCTCTGCCTGGCATGGAAGCGAGCTCCTTCCAGTCAAGCAGGGAAATCCTGGGTCTCGTGTCATCCCCAGGTCGGCTGTATGTTTCGTCACCTGCCCTCCAACTCTCCCTGGGCTGGGCTGGGGAGCGGGAGTTGTATGACTCTTGTCAGTCTTGGGCCTTCCCTAGTCCCACTGCCTCTGTTTCCCCTGAGTTTGATCTCTGTTAGGAGATTTCTAAGATTTCTGCATTTCTCCATCTCCGCATGGCCCTTGCCTTCTCCTCTTAGTTCGGAACCTCTGGACTGGAGGATGCTTCTGAGGGCATGGGGACCTGAGTTGGAGTGGGGAGGGGTGTTGACTCATCAACAGCATCATTTCTCCCAGGAGACGCCCGTAACTCATCCAAGGTCAGGACTACACACTGTCACGCACACAGGTACACTCACAGCTTTTATCTTCACGCTCCCTAACCTTGGCAAATTGTACAATTTCTTTGAAGCTCAGTTTCCTCATCTGTACAATGGGGAAAAGCATTAGATTTCATGAATTACTATAAGTAAAGTGTCCAATACAGTGCTTAGCACGTAATGAAGCCTCAATACAATGTAGTTATTCTCCATGCCCCACAAAGCTGGCATGCCTAGCCTCAGACCTACCATTTTTTGGGGTGCAGTAAGGCTTCCTGTCCACCATGTTCCCAGGGACATTGTACTGATGGGTGGAAAGGCAGGTCTAAAGGGGTCACGAAGTTCTGGGAGGTTAAGGGAACGAGGAAGGAGATTGAGCAACAAGGAAAGAGCTTGCCAAGAAGGAGGTGTGAATATTGGGACTGAGGAGGCAGCTTAGAGATGGGCAAGGGGGCAGTTCCAGGCAGAAATGGTTCGTGGAGGCAGAAGGTCCCTGGGAGAGGGAGCAGTCTGGAGGGTGGGGCAGGGGCGAGGAGGGGGAGGTGGGGAGACCCAGGACTGAGGAAGTAAACAAGGGGAGCGCCACCACAGAGGTGGAGAGGTGGAGGGTGCTGCTGCTGGGAATCAACCCCCTCAGACTTTCCACTGCGAAGCGAAACCGTAAGCCCTGGGGTGCGGGGGGCGGGCCGGGAGGAGGGGAAGTGGGGAAGGTGGAGGGAAGGCCGGGCACAGGGGTGAAGGCCC+1

TIGIT promoter (NFAT1 binding sequences were yellow highlighted, while ChIP primer specific sequences were red font):

-2938
GCTGCCGCAGGATGTTGCCCTTGATTCTAGGCAGAGACAGGAGGCATCAGACAGCTGTGACAGCCATGGAGCTCCCCTACCACTTCTGAGCCAGAGGGAAGACTTTGGAGAAGCAGAAGCCCAAAGACACTGCTGGGAGATTGGTCTGTGCTCTGGGAGAACCCTAGGTTGTGTTTCCCGTGTACAAGCCACCGATCTTGTGGAAGGTACCACCACTCCCAGCCCCGCTTGGCTGAGGGCTGCGGGCCACAGGTTTTCAGGACCGTGCTTGTGGTTGGAGGCTGTGGACTCAAGTGCGGCTGGCCCGGGACTTTCACAGGGCGCAGGGCGGGCTAGGGGAAGGCCCAGCTGTGAACTGATAAGGGGGTTTTACAGTCTAGGCCAGCACTTCTTCCCCTTTCACGGGCAAACGTGACATTCTTTTTCAGCACCTGTACTCACCCAGCTGCATCAAGGCGGACCAGACTTGTCCAACTCACAGCCCCAAAAGTCAGGGCAGAATAAAGCACAGTGGCTGATTTTAGCATTTTTAAAATAGAAATGTGAGTCAGCACCTGATGGACCTCACAGATCCCTGCTATGCCACGAGGGCTGCTGACTGGCCTTAAAAGCTCAGGGTGGCCCGGGGAGCTCTCAGAGCAGGCCTGCTTCTCACAGCTGAGCCGCTCAGTGCTGGGGGCTGGTGTTGAGTGGGTGGGAACCAGCCAGGTTCCCCGTGGAGTCTGTGCAGGAGGCTCTGGAGGGTCTGGGGTAGATTCCCTGGGGAGAGACTTCTGTCATTTGCTTTCCTCCAAGGCAGCCTCAGGGTCTAGCTGGCAAGTGGTCACTTTCCTGCAGAAACATGCTATCCTCCTGTTCTATCAGATAAGGAGGGCAGAATGAAGAGGTTGGGTGGAACCCTGGCCTTGGAGTCTGGAGACTAGGATTTTACTCTTGACAAAGCACATTATATAGATAGGGATATTGTCCTCCAATTTTCCTTTCACTTCCTTAATTAATATTGTTATCAATACTACTGTTGATGTATTGAACTCCTGCCATCTTCCAGGCACTGTGCTGAACACTTCACCAAACACTAGAGAATTAAATCCTCACAACCCTATGAAAGAGGTACTCTTATTCCTATATGCACATGGGGAAACTGAGCCTTCAGGAGGTTGAGAAAGTTTTCCAAGGTCACCAAACAGGATGACTTTGGACCCAGTCTGTTAATCATCCCCCTCTAGAGCACCATTGATCAAAATGGACTTGACCATATTCCTCTTTGGACTGGTTACTTAGCAAAAAACAGAAAAATTATTTAACAATTGTTTAAAACCACCTCTAAGTTTCTCTGGATATCTCATTATATAAGAGAGGTCTCACCTCTTTGGCTCCCACCCAGCTCAACTATGGAAACACAGGCTGTAAATTTTTATAGCCCAAATCCTAGTGTCCACCACTTTCTGTGCTCTAGTCCCACTAGCTGCTCTTGGCCCTGCCACTCCTGGCTCCCCGGCCTTAGTGAGTTTGGGAAACATCCCTCCAGTTCAACCTTGGCACCTTCCCTTGGACACACATTAGGTATTATTTCTCCTTCAGCTGAAGGCTTTGTAGCTATTGAAAGGCAAGGCATCTCTTGATGTGAGCCTTGAAGGACAGGTTGCTTTGGGTAGGCAGAGAGAAGCTGGTAAGGCGTATATTGTCTAAGGGTCTGGAGGTGGGGTATAAGGGGTATACAAGTCATAGAGTTGGGGATAGGCAGAACCCAGAGAGGGGAGAAAACTGGCCTGAGATAGAAGACCTTGCAAGGGAGAAGGAGGGTAGGAGCCTGGATGGTGTGAAATGCTAGAGGTGCATGGCTGGAATGGAGGGGGTGGGGCACCAGGCTTCAGATGCTTCTGGGTGCAGATGCAGATTTTCAGGATGTACAACATGGAGCATCCCCAAGGCAACTGTGTTGAGAGCCTGCGAGGCAGGTGAATGTGGATCTTCTACCCCCTCTCCTGACTGAGTTCACCAAGGAACAAGCTTTGTAAACTATTTGAGGGTAGGGGCTGTGATTATTTACTCTCATATCCTCAGAGCCTGGTGTTGAGGTTGGTGCTTTGTAGGCACCCAGGGACTTTCAAATGAATGAAGGGAGGGAGGGAGGAAAGAAGGATGGGTCCATAGTAGGACCTGGTGATGGGCTGGGAGCTCCAGGCAAATGTCAACCAATCCCTCTCCTGGGTCAGCTCCCAGGGGCTCACCCTTCTTTGCATTTCCAGCTCTCATGAGGTCATTGTGCACAGGAAAGCTCTCTCCTCTAATCTCCTCTGATCCTACTGCACCAGAGAAATCAAGCCAGAATTCAATAAAGTCTCAGTCCAGATAAACAAGACAAAAGAAATAAGATTCGAGTAGAAGATCTCCTTCAAGGGAAAGTTGCTGTGTTTGTCCAAGACCTTTGTCCCATCCATGTATCATCCCCCAAGTAAACACTTCTTGTTCACCTGTTCATTAGATTTCAAGTGCAGTCCCTGGCCTGTAAGTCCCTACAATGATAAGCTTCTCTTATCATTGCACATTCTTCATCAGGAGGATGCCAGAGGAGCTCAGCCAACAGTTCCTCATCAGTAGCAGATTCTTCAGAATCTTGGGCACTACACAGATGCCCTTGAGCTCTTTGAATAAAGGCTGATTTTTAGAAAAAACATTAAGACAGAACTTAAAAACAATAGATTGACTATAATCCAAAGACGAGTGTACCTCTAACCACAATTTTCATTTATTTTTAAATGTTTCCTTCATGGCCTTTCTTGTGGCTCACCCTATGCAGTTTGTGTATTTGTTGACAACTTTATGTGTTTTTAATATGGTTTTTGCCAAACTTGGTTTTTCCGAGACCGTCTTTTCTCAGAGGCTCAGTTTTACCGTCCTATCTGCAGTCGGCTACTTTCAGTGGCAGAAGAGGCC+1

TNF promoter (ATF2/c-Jun binding sequence was yellow highlighted, while ChIP primer specific sequences were red font):

-202 ATGCTTGTGTGTCCCCAACTTTCCAAATCCCCGCCCCCGCGATGGAGAAGAAACCGAGACAGAAGGTGCAGGGCCCACTACCGCTTCCTCCAGATGAGCTCATGGGTTTCTCCACCAAGGAAGTTTTCCGCTGGTTGAATGATTCTTTCCCCGCCCTCCTCTCGCCCCAGGGACATATAAAGGCAGTTGTTGGCACACCCA+1
